# Inborn errors of immunity with susceptibility to *S. aureus* infections

**DOI:** 10.3389/fped.2024.1389650

**Published:** 2024-04-24

**Authors:** Hannah Kurz, Kai Lehmberg, Susan Farmand

**Affiliations:** ^1^ Department of Pediatrics, University Medical Center Hamburg-Eppendorf, Hamburg, Germany; ^2^ Division of Pediatric Stem Cell Transplantation and Immunology, Department of Pediatric Hematology and Oncology, University Medical Center Hamburg-Eppendorf, Hamburg, Germany

**Keywords:** *S. aureus*, inborn errors of immunity (IEI), immunodeficiency, STAT3 deficiency, neutrophil dysfunction, chronic granulomatous disease (CGD), neutropenia, IL-6 deficiency

## Abstract

*Staphylococcus aureus* (*S. aureus*) is a significant human pathogen, in particular in patients with an underlying medical condition. It is equipped with a large variety of virulence factors enabling both colonization and invasive disease. The spectrum of manifestation is broad, ranging from superficial skin infections to life-threatening conditions like pneumonia and sepsis. As a major cause of healthcare-associated infections, there is a great need in understanding staphylococcal immunity and defense mechanisms. Patients with inborn errors of immunity (IEI) frequently present with pathological infection susceptibility, however, not all of them are prone to *S. aureus* infection. Thus, enhanced frequency or severity of *S. aureus* infections can serve as a clinical indicator of a specific underlying immunological impairment. In addition, the analysis of immunological functions in patients with susceptibility to *S. aureus* provides a unique opportunity of understanding the complex interplay between staphylococcal virulence and host immune predisposition. While the importance of quantitatively and qualitatively normal neutrophils is widely known, less awareness exists about the role of specific cytokines such as functional interleukin (IL)-6 signaling. This review categorizes well-known IEI in light of their susceptibility to *S. aureus* and discusses the relevant associated pathomechanisms. Understanding host-pathogen-interactions in *S. aureus* infections in susceptible individuals can pave the way for more effective management and preventive treatment options. Moreover, these insights might help to identify patients who should be screened for an underlying IEI. Ultimately, enhanced understanding of pathogenesis and immune responses in *S. aureus* infections may also be of relevance for the general population.

## Introduction

The current list of inborn errors of immunity (IEI) comprises more than 485 monogenetic gene defects (1). Enhanced susceptibility to a specific pathogen such as *Staphylococcus aureus* (*S. aureus)* may raise suspicion of a certain type of immunological impairment. *Staphylococcus aureus* is a great challenge to our health care systems (2). Despite being considered a commensal, with a colonization rate of 20%–30% in the healthy population (3), it can also cause a wide variety of different infections. It is a leading cause of skin and soft tissue infections and abscesses, but may also lead to lung infections, osteomyelitis or endocarditis, in particular in patients with underlying conditions (2). The ability to colonize but also to cause harm to the host, emerges from a complex interaction between the pathogen and its host (4). *Staphylococcus aureus* is a specialist in adapting to the human host by evading almost every aspect of the immune system (5). In the last decades, changes in strains have led to an increase of *S. aureus* infections in otherwise healthy individuals (6). Thus, staphylococcal defense in the individual is shaped by both pathogen virulence factors as well as the patient's immune predisposition (4). Recurrent or severe *S. aureus* infections may both be an indicator of certain IEI and specific IEI can teach us about essential immune functions for staphylococcal defense.

### *S. aureus* immune evasion and host immune response

**Figure 1:**
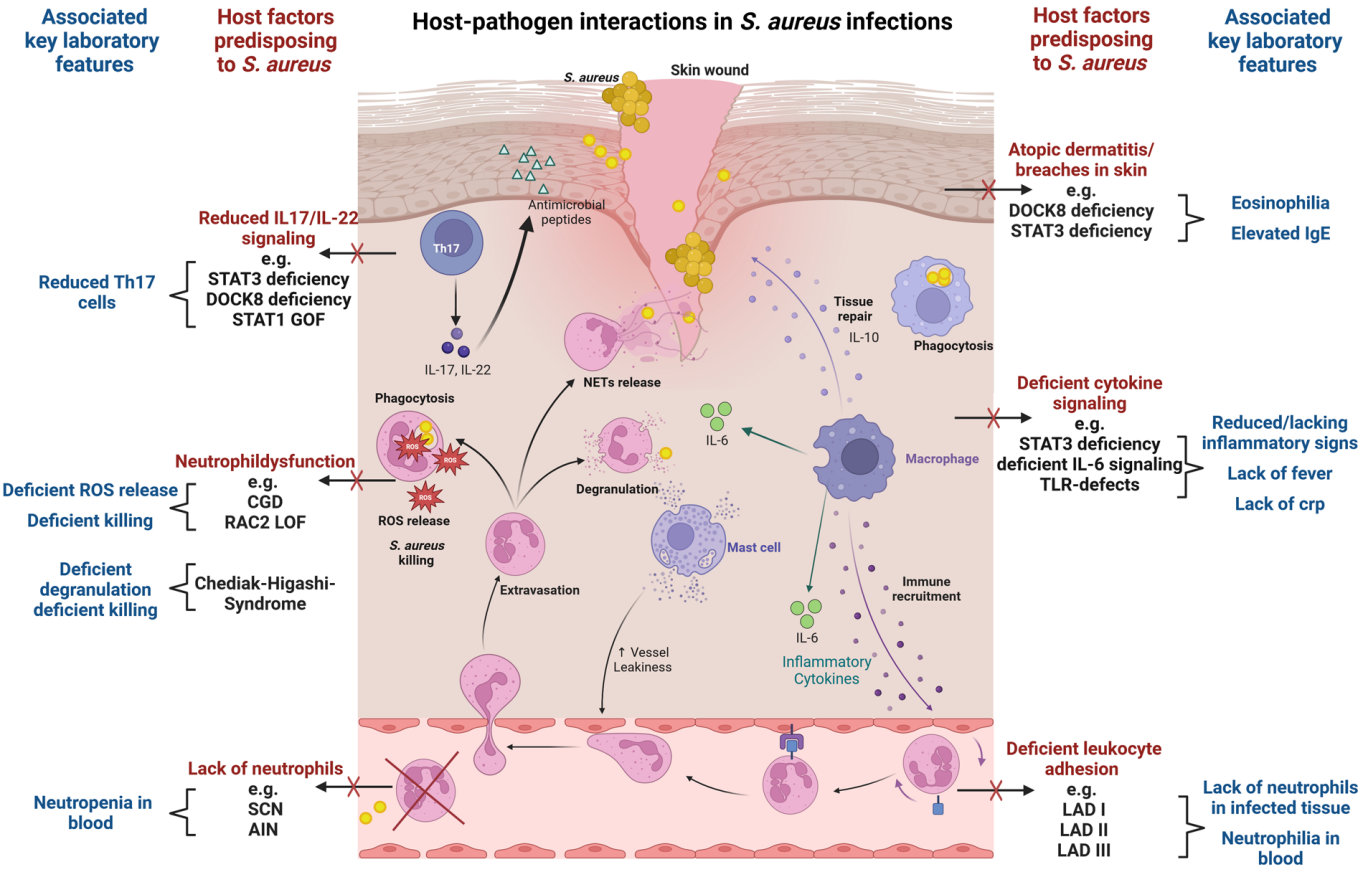
Host-pathogen interactions in *S. aureus* infections. The figure visualizes key immunological defense mechanisms and highlights host factors predisposing to *S. aureus* infection in case of deficiency. Commonly associated laboratory findings in the respective setting are also displayed. Selective examples of IEI with susceptibility to *S. aureus* infection are provided. The figure provides a simplified overview, and displayed host factors and interactions do not claim to be complete. The figure was created with BioRender.com.

Staphylococcal infections often arise from asymptomatic colonization and breaches through skin and mucosal barriers (7) (Figure 1). Immune evasion strategies of *S. aureus* are abundant and tackle particularly innate immunity (8, 9). Examples include inhibition of immune recognition, prevention of complement activation (10), resistance to phagosomal killing (5) and direct killing of immune cells through different leucocidins (7). In addition, presence of peptidoglycan layer, polysaccharide capsule and surface proteins hamper opsonization (7). The most important players in *S. aureus* defense are phagocytes. In particular neutrophils, along with tissue-resident or monocyte-derived macrophages, are instrumental in identifying, engulfing, and eliminating staphylococci (11). As the first line of innate cellular defense, they also orchestrate subsequent immune responses. The crucial role of neutrophils is clearly evidenced by the enhanced staphylococcal susceptibility of patients with numeric or functional neutrophil defects (12, 13). *Staphylococcus aureus* has developed numerous mechanisms to reduce neutrophil extravasation, activation, and chemotaxis (9), and may also evade neutrophil extracellular traps using nucleases and proteases (14). Secretion of exopolysaccharides and biofilm formation inhibit phagocytosis (7). When internalized by phagocytes, *S. aureus* may neutralize reactive oxygen species and employ enzymes for survival (8). Through intracellular survival both in phagocytic and non-phagocytic cells, *S. aureus* may evade antibiotic killing and facilitate subsequent dissemination (15). Induction of IL-10 by *S. aureus* may lead to a phenotypic switch in the immune response during persistent staphylococcal infection allowing its persistence as commensal (16). Toxins like Panton–Valentine leucocidin (PVL), which are harbored by some more virulent strains, destroy immune cells and may lead to treatment failure and severe infections even in immunocompetent patients (17, 18). While most virulence factors address innate immunity, *S. aureus* may also interfere with the adaptive immune response, using proteins like SpA to bind immunoglobulins (19) and superantigens like TSST-1 to induce cytokine release and toxic shock syndrome (20).

The evasion strategies of *S. aureus* challenge infection management, prevention and vaccine development (8). We provide an overview of IEI that render individuals susceptible to *S. aureus* infections (Table 1 and Supplementary Table S1), highlighting key immunological defense mechanism involved in staphylococcal immunity.

**Table 1:** IEI with recurrent or severe *S. aureus* infections.

Immune dysfunction	Underlying defect of immune dysfunction	Selected examples	Typical type of *S. aureus* infection	Severity of *S. aureus* infection	Additional typical infections, suggestive signs	Prophylaxis options	General treatment options
IEI with neutropenia	Severe congenital neutropenia (SCN)	Neutrophil elastase defects, HAX1 def., X-linked neutropenia (WAS), Shwachman-Diamond syndrome, etc.	Blood stream, organ infections, abscesses	Severe, invasive, rapidly progressing	Fungal infections (*Candida, Aspergillus*), gram negative bacterial infections, gingivitis, oral ulcers	G-CSF, TMP/SMX, antifungal	Rapid empiric antibiotic treatment, increase G-CSF during infections, HSCT in some SCN
	Autoimmune neutropenia (AIN)	"benign” AIN (infancy), AIN associated to underlying IEI/autoimmune diseases	Skin infections/folliculitis, rarely invasive infections	Mild-moderate	AIN of infancy: rarely other signs, AIN >5 years investigate for underlying IEI/Autoimmune disease	Rarely needed	Antibiotic treatment as needed, G-CSF only in selected cases
	Combined IEI with Neutropenia	e.g., CD40l or CD40deficiency, RAC2 GOF, PGM3 deficiency	Blood stream, organ infections	Severe, invasive, rapidly progressing	Opportunistic infections (*PJP, Aspergillus*). Depending on underlying IEI systemic features	TMP/SMX, antifungal, IVIG/ScIG	Rapid empiric antibiotic treatment, G-CSF, HSCT
IEI with neutrophil dysfunction	Deficient release of ROS to all stimuli/deficient killing	Chronic granulomatous disease (CGD): different traits	Abscesses (skin, organ), pneumonia, blood stream infections	Moderate to severe	Fungal infections (*Candida, Aspergillus*), gram negative, Catalase pos (e.g., *Nocardia, Burkholderia, Serratia*). Granuloma, colitis	TMP/SMX, antifungal	Rapid empiric antibiotic treatment, INF-y, HSCT, gene therapy
	Deficient ROS release/neutrophil chemotaxis (fMLP)	RAC2 LOF	Abscesses with lack of pus	Moderate to severe	Delayed wound healing and omphalitis	TMP/SMX	Antibiotic treatment, otherwise not well defined, depending on severity
	Deficient rolling, adhesion, extravasation	3 different types: LAD I, LAD II, LAD III	Skin and soft tissue infections with lack of pus	Moderate to severe in LAD I and III, milder in LAD II	Delayed wound healing and omphalitis in LAD I and LAD III. Neurodevelopmental impairment in LAD II. Bleeding in LAD III	TMP/SMX	Antibiotic treatment, fucose-based therapy for LAD II, HSCT (LAD I and LAD III)
	Deficient degranulation, reduced bactericidal activity	Chediak-Higashi-Syndrome	Skin infections	Moderate to severe (in particular if neutropenic)	Recurrent pyogenic infections. Systemic features: oculocutaneous albinism, neurological features, HLH.	G-CSF if neutropenic	HSCT, in particular if profound defects in cytotoxicity
IEI with defective cytokine signaling/TLR-defects	Reduced STAT3 signaling (incl. low IL-6 signaling)	STAT3-deficient HIES, AR ZNF341	Skin infections/cold abscesses, pneumonia	Severe tissue destruction possible, there may be an inadequate inflammatory response related to the degree of defective IL-6 signaling (potential lack of fever/low or absent CRP)	CMC. Multisystemic features, eczema, eosinophilia, IgE elevation, low Th17, encapsulated bacteria.	TMP/SMX, antifungal, IVIG/ScIG (STAT3-def.)	Treat with antibiotics if infection is suspected independent of CRP/fever/general conditions. Surgical abscess drainage may be required
	Reduced IL-6 family signaling	AR partial LOF IL6ST or AD DN IL6ST			Skeletal abnormalities, eczema, eosinophilia, IgE elevation, variable Th17 cells	Consider TMP/SMX	
	Reduced IL-6 signaling only	IL6 receptor deficiency (AR IL6R def (IL6R)			Atopic dermatitis, eosinophilia, IgE elevation, normal Th17		
		IL-6 autoantibodies	Skin and soft tissue infections, sepsis in 1 case reported		Lack of multisystemic features no eczema, no CMC		
	TLR-signaling defects	IRAK-4 def, MyD88 def, EDA-ID (XR NEMO-def. and AD IKBA GOF)	Severe, invasive pyogenic infections (meningitis, sepsis, osteomyelitis,..). Skin infections	Rapidly progressing, severe infection, but lack of inflammation	Severe infection susceptibility to *S. pneumoniae. P. aeruginosa* also frequent. Additional viral/mycobact. infections, colitis and ectodermal dysplasia in EDA-ID	TMP/SMX+penicillin, Vaccination, in particular against encapsulated bacteria, IVIG/ScIG	Rapid empiric parenteral antibiotic treatment independent from CRP, fever or general conditions. Consider HSCT

The table lists the most prominent examples of IEI with susceptibility to *S. aureus* infections. IEI are grouped according to their most relevant underlying immune dysfunction associated to staphylococcal susceptibility. Descriptions of different IEI are kept to a minimum, and only key findings suggestive of the respective IEI are highlighted (for more details and references also see Supplementary Table S1). The table also displays potential prophylaxis and treatment options. In general, TMP/SMX may be used in patients with significant disease as antibiotic prophylaxis. However, local epidemiology and individual antimicrobial resistance needs to be considered. In IEI with deficient cytokine signaling, but also in patients with neutropenia, there is an urgent need to treat with empiric antibiotics upon clinical suspicion of infection regardless laboratory inflammation markers. HSCT can be a curative option in some diseases. Still, the need to treat may be variable for different genetic variants and also depends on the clinical severity.

### IEI with low neutrophil numbers and susceptibility to *S. aureus* infections

**Severe congenital neutropenia (SCN)** is usually characterized by severe neutropenia (<500/µl) due to myeloid maturation arrest in the bone marrow. Over 20 different genes have been identified (21). Lack of mature neutrophils leads to a severe infectious phenotype with potentially life-threatening disease in the first months of life. Infections are caused not only by *S. aureus* but also by gram negative bacteria, and blood stream infections are common. Depending on the underlying gene defect there may be additional somatic features (Supplementary Table S1) (22).

Primary **autoimmune neutropenia (AIN)** of infancy, which is the most common type of neutropenia in childhood and may also present with nearly absent neutrophils and susceptibility to staphylococcal skin infections (abscesses, furunculosis), needs to be separated from SCN. AIN is typically detected in infancy, frequently as an incidental finding, and shows spontaneous remission in early childhood (23). Neutrophils mature normally in the bone marrow but peripheral numbers may be very low due to the presence of anti-neutrophilic antibodies. Infections are less severe compared to SCN. While the detection of anti-neutrophilic antibodies is suggestive of AIN it does not fully exclude additional SCN. Thus, in cases with severe infections or persistent neutropenia bone marrow evaluation and genetic testing may be indicated. If detected in older children or adults, AIN is more likely to be an immune phenomenon related to another IEI/autoimmune disorders requiring further diagnostic workup (24).

### IEI with neutrophil function defects and susceptibility to *S. aureus* infections

**Chronic granulomatous disease (CGD)** represents the most common hereditary phagocyte dysfunction with an estimated prevalence of around 1:200,000 (25, 26). CGD leads to deficient reactive oxygen species (ROS) generation due to loss-of-function mutations affecting different aspects of the multicomponent enzyme NADPH oxidase in phagocytes (Nox2) (27). CGD patients experience severe infections accompanied by granuloma and abscess formation. *Staphylococcus aureus* is the most common pathogen isolated from skin infections/abscesses, liver abscesses and lymphadenitis, but it may also lead to pulmonary infections or sepsis. Patients are also very susceptible to *Aspergillus spp.* (26). Other characteristic pathogens in CGD include gram negative bacteria (e.g., *Salmonella*) and catalase positive bacteria (e.g., *Burkholderia*, *Serratia* and *Nocardia)* (12, 28). Additionally, CGD is associated with inflammatory complications like colitis, which might be related to defective T-cell regulation but also hyperactivation of NF-kB and inflammasome pathways (27, 29).

**Leukocyte adhesion deficiency (LAD)** is characterized by functional defects in neutrophil adhesion, integrin activation or rolling, leading to an inability to migrate effectively to infection sites (30). This results in a striking discrepancy with lack of pus formation at infection sites despite significant leukocytosis with neutrophilia in the blood. LAD patients typically experience recurrent bacterial and fungal infections, delayed wound healing, and other associated features (31). Three different genetic defects affecting neutrophils are known. Associated features are omphalitis and gingivitis (LAD I), developmental impairment and short statue (LAD II), and bleeding tendency (LAD III) (30, 32).

### Combined IEI which frequently cause neutropenia or neutrophil dysfunction

Neutropenia has also been described in certain combined immunodeficiencies. Typical examples are **CD40Ligand (CD40l)** and **CD40 deficiency**, which are characterized by abnormal serum immunoglobulin levels due to impaired interaction between CD40l on T cells and CD40 on antigen-presenting cells (33, 34). These conditions lead to both impaired cellular and humoral immunity, which results in a broad infection phenotype. Patients frequently present with opportunistic infections (e.g., *pneumocystis jirovecii*, *cryptosporidium*, *aspergillus spp*.) (35). IgM may be elevated concomitantly to low IgA and IgG, which lead to bacterial respiratory and gastrointestinal infections (33). Intermittent or permanent neutropenia might be related to deficient release of growth factors important for granulopoiesis due to impaired CD40-CD40l-interaction (36). Furthermore, functional defects in neutrophils have been described in CD40l deficiency (37).

Mutations in Ras-related C3 botulinum toxin substrate 2 (RAC2) are also typically affecting neutrophil function. RAC2 is an essential regulator of neutrophil chemotaxis and contributes to NADPH oxidase function (38). Autosomal-dominant (AD) **RAC2 loss of function (LOF)** mutations cause LAD-like disease with neutrophilia and functional neutrophil defects (e.g., deficient chemotaxis and ROS generation) (39). In contrast, AD **RAC2 gain of function (GOF)** mutations lead to (severe) combined immunodeficiencies with lymphopenia and low immunoglobulins, frequent neutropenia and functional neutrophil abnormalities (38, 40).

Neutropenia has also been reported in some patients with deficiency in phosphoglucomutase 3 (PGM3), a disorder of glycosylation which is currently classified as autosomal-recessive Hyper IgE syndrome (1). **PGM3 deficiency** presents with eczema, eosinophilia, elevated IgE, but may also display a CID/SCID phenotype, facial dysmorphism and neurocognitive impairment (41).

Patients with autosomal-recessive deficiency of dedicator of cytokines (DOCK) 8 display severe atopic dermatitis with *S. aureus* colonization and skin infections (**DOCK8 deficiency**). Osteomyelitis has also been reported (42). DOCK8 plays a crucial role in lymphocyte proliferation, migration of dendritic cells, and generation of long-term memory in B- and T cells, thus predisposing patients to a mostly severe phenotype regarding viral and mycobacterial infections (43). Dysfunction of regulatory T-cells together with *S. aureus* exposure have been suggested to drive severe eczema in DOCK8 deficiency (44) and DOCK8-deficient murine neutrophils were prone to undergo *S. aureus*-induced cell death (45). In addition, reduced signal transducer and activator of transcription 3 (STAT3) signaling and low T helper 17 (Th17) cells have also been reported (46).

### IEI with staphylococcal susceptibility associated to defective cytokine signaling

Autosomal-dominant Hyper-IgE syndrome due to dominant-negative mutations in STAT3 (**STAT3-HIES**) is one of the key IEI associated with a specific susceptibility to *S. aureus* infections, particularly in the skin and lung (47). Recurrent “cold” abscesses with lacking systemic signs of infections are typical. STAT3 functions as a transcription factor downstream of the tyrosine kinases janus activated kinase (JAK)1, JAK2, and tyrosine kinase 2 (TYK2) and enables signal transduction through various cytokines, such as interleukin-6 (IL-6), IL-10, IL-11, IL-21, and IL-23 (48). STAT3 deficiency results in failure of Th17 cell differentiation (49). Th17 function has been shown to be pivotal in Candida defense (50), explaining the patients' predisposition to mucocutaneous candidiasis. Th17 cells aid epithelial cells to produce neutrophil-recruiting chemokines and antimicrobial factors such as ß-defensins, which may be relevant for staphylococcal defense (51). STAT3-deficient neutrophils display normal functions (52), but are prone to undergo *S. aureus*-induced cell death (53). Furthermore, STAT3-HIES patients display variable antibody responses and low numbers of memory B cells, which likely contributes to enhanced incidence of respiratory infections with *H. influenzae* and *S. pneumoniae* (52). STAT3 is ubiquitously expressed and multisystemic features are present. Thus, deficient epithelial STAT3 signaling may contribute to aberrant staphylococcal control by cytokine dysregulation and aberrant tissue remodeling (54, 55). STAT3 is involved in both pro- and anti-inflammatory signaling which complicates our understanding of single factors for the overall phenotype.

Autosomal-recessive **ZNF341 deficiency** leads to reduced cytokine signaling via STAT3 and resembles STAT3-HIES by displaying similar multisystemic features (e.g., bone fractures, retention of primary teeth, facial dysmorphism) but also staphylococcal infections (56).

IEI affecting single cytokines may teach us about their individual contribution. Lack of functional IL-6 cytokine family signaling reduces typical local inflammatory reaction, leads to low CRP and reduced systemic symptoms although tissue damage may be considerable. Defective IL-6 signaling either by **IL-6 receptor deficiency** (57) or by partial **IL-6 signal transducer deficiency** (IL6ST) (58) also leads to pyogenic infections, cold abscesses and pulmonary *S. aureus* infections. Additionally, phenocopies of IEI such as autoantibodies against IL-6 show increased susceptibility to *S. aureus* infection lacking CRP response (59). *Staphylococcus aureus* infections are also described in ERBIN deficiency which recapitulates some features of STAT3 deficiency (60).

Frequent *S. aureus* skin infections have also been reported in patients with **STAT1GOF** who are very susceptible to fungal infections, have low Th17 cells, and display a high rate of autoimmune features (61, 62).

### IEI with defects in toll-like receptor (TLR)-signaling and susceptibility to *S. aureus*

Autosomal-recessive **IRAK-4** and **MyD88 deficiencies** affect TLR and IL-1R induced activation of NF-κB and MAPKs through the classical pathway (63). They disrupt key pathways in the innate immune response and usually present with bacterial pyogenic infections early in life (<2years of age). Most common pathogens are *S. pneumoniae*, *S. aureus and Pseudomonas aeruginosa* (64). Lack of TLR-induced signaling affects particularly the production of IL-6 and IL-8, and may lead to severe invasive infections (e.g., meningitis, sepsis, osteomyelitis, arthritis and abscesses), but also localized skin infections, lymphadenitis and ENT infections, usually without marked fever or increase of CRP (64). Still, pus is seen at the site of infection, which underlines that pus formation is not dependent on TLR-related cytokine signaling. As signs of infections may be absent but invasive infection may be rapidly progressing, it is vital to initiate empirical antibiotic treatment as soon as infection is suspected (64).

**NEMO deficiency** and **IκBα GOF**, which affect both NF-κB and TRIF-dependent signaling, result in a broad spectrum of immune dysfunctions and present also typically with colitis and ectodermal dysplasia. Apart from pyogenic bacterial infections, patients may also display mycobacterial infections, severe viral infections and opportunistic infections (64). Recently, more rare genetic defects associated to TLR-signaling have been reported, with variable phenotype depending on the protein involved.

### Other diseases with susceptibility to *S. aureus*

Apart from classical IEI, increased susceptibility to *S. aureus* infections has also been reported in diseases such as **cystic fibrosis**, **HIV infection** and/or **diabetes mellitus** (65–68). In addition to aberrant host immune response, susceptibility to *S. aureus* may also be enhanced by colonization of multi-resistant strains (MRSA) carrying specific virulence factors.

## Discussion: controversies, current knowledge gaps and future perspectives

While the key role of innate immunity for staphylococcal defense is well-established, the contribution of adaptive immunity is less clear.

In regards to B-cell immunity, evidence for a protective role of *S. aureus* antibodies is scarce. In fact, it has lately been suggested that *S. aureus* may induce non-protective antibodies, which then interfere with protective immune responses (69) facilitating commensalism and recurrent infections. Furthermore, patients with antibody deficiency do not display a specifically enhanced susceptibility to *S. aureus,* while they are clearly susceptible to other bacteria with a polysaccharide capsule (e.g., *S. pneumoniae, H. influenzae*). In contrast to the successful vaccine development for other encapsulated bacteria, there is still no available vaccine against *S*. *aureus*, and even adequate antibody induction to relevant *S. aureus* virulence factors did not lead to protection (70). The ability of anti-TSST-1 antibodies to provide protective immunity against superantigen-driven toxic shock syndrome appears to be an exception to the above, with IVIG being used as potential adjunctive therapy to ameliorate the symptoms (71).

Regarding the relevance of T cells, Th17 cells are often suggested to contribute to anti-staphylococcal-response, particularly at mucosa and skin sites (51). In mice, several studies document the importance of functional IL-17 signaling for the protection against mucocutaneous *S. aureus* infections (72, 73). Patients with IL-17RA deficiency are very prone to mucocutaneous candidiasis but do also display staphylococcal skin infections (74, 75). The initial hypothesis regarding the relevance of Th17 cells to prevent staphylococcal skin infection is closely related to the observed lack of Th17 in STAT3 deficiency (51). While the role of Th17 for candida defense is supported by other IEI with specifically deficient IL-17 signaling such as IL-17 autoantibodies (75), their relevance for *S. aureus* infections appears less significant. In the context of STAT3-HIES, the abundant changes in different cytokine signaling pathways and the contribution of ubiquitously deficient STAT3 needs to be considered. Of note, deficient IL-6 cytokine signaling is sufficient to predispose to staphylococcal infection even in the setting of normal Th17 cells (58, 76), and mere lack of Th17 cells does not induce susceptibility to *S. aureus* infection as evidenced in patients with IL12B/IL12RB1 deficiency (77) or CARD9 deficiency (78). Notably, STAT3-deficient patients with somatic mosaicism and normal Th17 compartment may still present with boils and pneumonia (79). Thus, lack of IL-17 signaling alone is likely insufficient in explaining enhanced susceptibility to *S. aureus,* even though patients may be more prone to folliculitis (74).

IEI with impairments in TLR and NF-*κ*B signaling pathways such as in IRAK-4 or MyD88 deficiency, underline the significance of these pathways in recognizing and responding to *S. aureus* (80). Patients with STAT3-HIES, ZNF341 deficiency, partial IL6ST deficiency and IL-6 receptor deficiency all share deficient IL-6 signaling and enhanced frequency of “cold” staphylococcal abscesses and lung infections (1). IL-6 is a pleiotropic cytokine that is vital for acute-phase responses, defense against bacterial infections and tissue regeneration (81). The shared phenotype argues for an essential role of IL-6 in staphylococcal defense (82). Still, the precise molecular mechanism behind this particular predisposition and the contribution of other pathways is unknown.

Complement deficiencies might serve as additional risk factors in the context of *S. aureus* infections due to the crucial role of the complement system in opsonizing pathogens and facilitating their clearance by phagocytes. Susceptibility to *S. aureus* infections has been described in patients with C2 and C3 deficiencies (83) and complement activation was found to reduce persistent intracellular *S. aureus* burden in keratinocytes (84). Still, the role of complement in the defense against this pathogen appears less pronounced compared to its critical function in combating other encapsulated bacteria.

More recently, it has been proposed that specific genes may predispose to more severe infections via impairment of selective immune defense mechanism such as the altered response of non-leukocytic cells to staphylococcal alpha-toxin in OTULIN haploinsufficiency (85). With the growing use of NGS our understanding of specific factors in staphylococcal immunity will likely expand further. Still, the rareness of single IEI may hamper reliability of certain genotype-phenotype associations. An example is TYK2 deficiency, where the originally identified patient with susceptibility to *S. aureus* and hyper-IgE phenotype (86) was later judged to display deficient IL-6 signaling unrelated to TYK2 deficiency (87).

Last, the ability of *S. aureus* to survive intracellularly, notably within neutrophils, macrophages and as small colony variants in epithelial cells, complicates the immune response and treatment strategies and might facilitate recurrent infections (88). Together with the multiple other evasion strategies this poses significant challenges in vaccine development against *S. aureus*. In the light of growing rates of MRSA, it therefore remains essential to continue to assess host-pathogen interactions on a functional level and further enhance our understanding about crucial immune defense mechanisms.

## Conclusion and diagnostic suggestions

➢ Basic immunological workup in patients with recurrent or severe *S. aureus* infections should include a differential blood count and IgG, IgA, IgM, IgE
➢ Specific testing for CGD, HIES, complement deficiency, LAD, TLR deficiency, exclusion of secondary immunodeficiencies and assessment for phenocopies of IEI as well as genetic analysis may be warranted
➢ Inconclusive immunological investigation should be complemented by assessment of staphylococcal colonization

## Supplementary material

### Abbreviations

AD, autosomal-dominant; AIN, autoimmune neutropenia; AR, autosomal-recessive; C2, C3, complement component 2, complement component 3; CARD9, caspase recruitment domain family member 9; CD40, cluster of differentiation 40; CD40l, cluster of differentiation 40 ligand; CGD, chronic granulomatous disease; CID, combined immunodeficiency; CRP, C-reactive protein; DOCK, dedicator of cytokinesis; G-CSF, granulocyte colony-stimulating factor; GOF, gain of function; HAX1, HCLS1 associated protein X-1; HIES, hyper IgE syndrome; HIV, human immunodeficiency virus; HSCT, hematopoietic stem cell transplantation; IEI, inborn errors of immunity; Ig, immunoglobulin; IL, interleukin; IL-17RA, IL-17 receptor A; IL12B, IL12RB1, IL-12 subunit beta, IL-12 receptor beta 1; IL6ST, IL-6 signal transducer; IRAK-4, IL-1 receptor-associated kinase 4; IVIG, intravenous immunoglobulin; JAK, Janus kinase; LAD, leukocyte adhesion deficiency; LOF, loss of function; MAPK, mitogen-activated protein kinase; MRSA, methicillin-resistant *Staphylococcus aureus*; MyD88, myeloid differentiation primary response 88; NADPH, nicotinamide adenine dinucleotide phosphate; NEMO, NF-κB essential modulator; NF-kB, nuclear factor kappa B; NGS, next generation sequencing; Nox2, NADPH oxidase 2; OTULIN, OTU deubiquitinase with linear linkage specificity; PGM, phosphoglucomutase; PVL, panton-valentine leucocidin; RAC2, ras-related C3 botulinum toxin substrate 2; ROS, reactive oxygen species; *S. aureus, Staphylococcus aureus;* SCID, severe combined immunodeficiency; SCN, severe congenital neutropenia; SpA, staphylococcal protein A; spp, species pluralis; STAT3, signal transducer and activator of transcription 3; STAT1, signal transducer and activator of transcription 1; Th17, T helper 17; TLR, toll-like receptor; TMP/SMX, trimethoprim/sulfamethoxazole; TRIF, TIR-domain-containing adapter-inducing interferon-β; TSST-1, toxic shock syndrome toxin-1; TYK2, tyrosine kinase 2; ZNF341, zinc finger protein 341.

## References

[1] Tangye SG Al-Herz W Bousfiha A Cunningham-Rundles C Franco JL Holland SM Human inborn errors of immunity: 2022 update on the classification from the international union of immunological societies expert committee. J Clin Immunol. (2022) 42(7):1473–507. 10.1007/s10875-022-01289-3 35748970 PMC9244088

[2] Tong SY Davis JS Eichenberger E Holland TL Fowler VG Jr. *Staphylococcus aureus* infections: epidemiology, pathophysiology, clinical manifestations, and management. Clin Microbiol Rev. (2015) 28(3):603–61. 10.1128/CMR.00134-14 26016486 PMC4451395

[3] Wertheim HF Melles DC Vos MC van Leeuwen W van Belkum A Verbrugh HA The role of nasal carriage in *Staphylococcus aureus* infections. Lancet Infect Dis. (2005) 5(12):751–62. 10.1016/S1473-3099(05)70295-4 16310147

[4] Kwiecinski JM Horswill AR. *Staphylococcus aureus* bloodstream infections: pathogenesis and regulatory mechanisms. Curr Opin Microbiol. (2020) 53:51–60. 10.1016/j.mib.2020.02.005 32172183 PMC7244392

[5] Thammavongsa V Kim HK Missiakas D Schneewind O. *Staphylococcal* manipulation of host immune responses. Nat Rev Microbiol. (2015) 13(9):529–43. 10.1038/nrmicro3521 26272408 PMC4625792

[6] David MZ Daum RS. Community-associated methicillin-resistant *Staphylococcus aureus*: epidemiology and clinical consequences of an emerging epidemic. Clin Microbiol Rev. (2010) 23(3):616–87. 10.1128/CMR.00081-09 20610826 PMC2901661

[7] Cheung GYC Bae JS Otto M. Pathogenicity and virulence of *Staphylococcus aureus*. Virulence. (2021) 12(1):547–69. 10.1080/21505594.2021.1878688 33522395 PMC7872022

[8] Wójcik-Bojek U Różalska B Sadowska B. *Staphylococcus aureus*-a known opponent against host defense mechanisms and vaccine development-do we still have a chance to win? Int J Mol Sci. (2022) 23(2). 10.3390/ijms23020948 PMC8781139 35055134

[9] de Jong NWM van Kessel KPM van Strijp JAG. Immune evasion by *Staphylococcus aureus*. Microbiol Spectr. (2019) 7(2). 30927347 10.1128/microbiolspec.gpp3-0061-2019 PMC11590434

[10] Loh JM Aghababa H Proft T. Eluding the immune system’s frontline defense: secreted complement evasion factors of pathogenic gram-positive cocci. Microbiol Res. (2023) 277:127512. 10.1016/j.micres.2023.127512 37826985

[11] Miller LS Cho JS. Immunity against *Staphylococcus aureus* cutaneous infections. Nat Rev Immunol. (2011) 11(8):505–18. 10.1038/nri3010 21720387 PMC5868361

[12] Marciano BE Spalding C Fitzgerald A Mann D Brown T Osgood S Common severe infections in chronic granulomatous disease. Clin Infect Dis. (2014) 60(8):1176–83. 10.1093/cid/ciu1154 25537876 PMC4400412

[13] van den Berg JM Kuijpers TW. Educational paper: defects in number and function of neutrophilic granulocytes causing primary immunodeficiency. Eur J Pediatr. (2011) 170(11):1369–76. 10.1007/s00431-011-1584-5 21968907 PMC3197933

[14] Berends ET Horswill AR Haste NM Monestier M Nizet V von Köckritz-Blickwede M. Nuclease expression by *Staphylococcus aureus* facilitates escape from neutrophil extracellular traps. J Innate Immun. (2010) 2(6):576–86. 10.1159/000319909 20829609 PMC2982853

[15] Löffler B Tuchscherr L Niemann S Peters G. *Staphylococcus aureus* persistence in non-professional phagocytes. Int J Med Microbiol. (2014) 304(2):170–6. 10.1016/j.ijmm.2013.11.011 24365645

[16] Li Z Peres AG Damian AC Madrenas J. Immunomodulation and disease tolerance to *Staphylococcus aureus*. Pathogens. (2015) 4(4):793–815. 10.3390/pathogens4040793 26580658 PMC4693165

[17] Missiakas D Winstel V. Selective host cell death by *Staphylococcus aureus*: a strategy for bacterial persistence. Front Immunol. (2020) 11:621733. 10.3389/fimmu.2020.621733 33552085 PMC7859115

[18] Rao Q Shang W Hu X Rao X. *Staphylococcus aureus* ST121: a globally disseminated hypervirulent clone. J Med Microbiol. (2015) 64(12):1462–73. 10.1099/jmm.0.000185 26445995

[19] Kobayashi SD DeLeo FR. *Staphylococcus aureus* protein A promotes immune suppression. mBio. (2013) 4(5). 10.1128/mbio.00764-13 PMC3791897 24085782

[20] Miethke T Duschek K Wahl C Heeg K Wagner H. Pathogenesis of the toxic shock syndrome: t cell mediated lethal shock caused by the superantigen TSST-1. Eur J Immunol. (1993) 23(7):1494–500. 10.1002/eji.1830230715 8325325

[21] Skokowa J Dale DC Touw IP Zeidler C Welte K. Severe congenital neutropenias. Nat Rev Dis Primers. (2017) 3:17032. 10.1038/nrdp.2017.32 28593997 PMC5821468

[22] Han X Lu S Gu C Bian Z Xie X Qiao X. Clinical features, epidemiology, and treatment of Shwachman-Diamond syndrome: a systematic review. BMC Pediatr. (2023) 23(1):503. 10.1186/s12887-023-04324-3 37803383 PMC10557232

[23] Bux J Behrens G Jaeger G Welte K. Diagnosis and clinical course of autoimmune neutropenia in infancy: analysis of 240 cases. Blood. (1998) 91(1):181–6. 10.1182/blood.V91.1.181 9414283

[24] Farruggia P Dufour C. Diagnosis and management of primary autoimmune neutropenia in children: insights for clinicians. Ther Adv Hematol. (2015) 6(1):15–24. 10.1177/2040620714556642 25642312 PMC4298488

[25] Buvelot H Posfay-Barbe KM Linder P Schrenzel J Krause KH. *Staphylococcus aureus*, phagocyte NADPH oxidase and chronic granulomatous disease. FEMS Microbiol Rev. (2017) 41(2):139–57. 27965320 10.1093/femsre/fuw042

[26] van den Berg JM van Koppen E Ahlin A Belohradsky BH Bernatowska E Corbeel L Chronic granulomatous disease: the European experience. PLoS One. (2009) 4(4):e5234. 10.1371/journal.pone.0005234 19381301 PMC2668749

[27] Roos D. Chronic granulomatous disease. Br Med Bull. (2016) 118(1):50–63. 10.1093/bmb/ldw009 26983962 PMC5127417

[28] Slack MA Thomsen IP. Prevention of infectious complications in patients with chronic granulomatous disease. J Pediatric Infect Dis Soc. (2018) 7(suppl_1):S25–30. 10.1093/jpids/piy016 29746681 PMC5946879

[29] Meissner F Seger RA Moshous D Fischer A Reichenbach J Zychlinsky A. Inflammasome activation in NADPH oxidase defective mononuclear phagocytes from patients with chronic granulomatous disease. Blood. (2010) 116(9):1570–3. 10.1182/blood-2010-01-264218 20495074 PMC2938844

[30] Hanna S Etzioni A. Leukocyte adhesion deficiencies. Ann N Y Acad Sci. (2012) 1250:50–5. 10.1111/j.1749-6632.2011.06389.x 22276660

[31] Roos D van Leeuwen K Madkaikar M Kambli PM Gupta M Mathews V Hematologically important mutations: leukocyte adhesion deficiency (second update). Blood Cells Mol Dis. (2023) 99:102726. 10.1016/j.bcmd.2023.102726 36696755

[32] Gazit Y Mory A Etzioni A Frydman M Scheuerman O Gershoni-Baruch R Leukocyte adhesion deficiency type II: long-term follow-up and review of the literature. J Clin Immunol. (2010) 30(2):308–13. 10.1007/s10875-009-9354-0 20099014

[33] Banday AZ Nisar R Patra PK Kaur A Sadanand R Chaudhry C Clinical and immunological features, genetic variants, and outcomes of patients with CD40 deficiency. J Clin Immunol. (2023) 44(1):17. 10.1007/s10875-023-01633-1 38129705 PMC11252661

[34] Ferrari S Giliani S Insalaco A Al-Ghonaium A Soresina AR Loubser M Mutations of CD40 gene cause an autosomal recessive form of immunodeficiency with hyper IgM. Proc Natl Acad Sci U S A. (2001) 98(22):12614–9. 10.1073/pnas.221456898 11675497 PMC60102

[35] Ferrua F Galimberti S Courteille V Slatter MA Booth C Moshous D Hematopoietic stem cell transplantation for CD40 ligand deficiency: results from an EBMT/ESID-IEWP-SCETIDE-PIDTC study. J Allergy Clin Immunol. (2019) 143(6):2238–53. 10.1016/j.jaci.2018.12.1010 30660643

[36] Mavroudi I Papadaki HA. The role of CD40/CD40 ligand interactions in bone marrow granulopoiesis. ScientificWorldJournal. (2011) 11:2011–9. 10.1100/2011/671453 22125452 PMC3217605

[37] Cabral-Marques O França TT Al-Sbiei A Schimke LF Khan TA Feriotti C CD40 ligand deficiency causes functional defects of peripheral neutrophils that are improved by exogenous IFN-γ. J Allergy Clin Immunol. (2018) 142(5):1571–88.9. 10.1016/j.jaci.2018.02.026 29518426 PMC6123297

[38] Hsu AP Donkó A Arrington ME Swamydas M Fink D Das A Dominant activating RAC2 mutation with lymphopenia, immunodeficiency, and cytoskeletal defects. Blood. (2019) 133(18):1977–88. 10.1182/blood-2018-11-886028 30723080 PMC6497516

[39] Ambruso DR Knall C Abell AN Panepinto J Kurkchubasche A Thurman G Human neutrophil immunodeficiency syndrome is associated with an inhibitory Rac2 mutation. Proc Natl Acad Sci U S A. (2000) 97(9):4654–9. 10.1073/pnas.080074897 10758162 PMC18288

[40] Donkó A Sharapova SO Kabat J Ganesan S Hauck F Marois L Clinical and functional spectrum of RAC2-related immunodeficiency. Blood. (2024). 10.1182/blood.2023022098 PMC11033590 38194689

[41] Fallahi M Jamee M Enayat J Abdollahimajd F Mesdaghi M Khoddami M Novel PGM3 mutation in two siblings with combined immunodeficiency and childhood bullous pemphigoid: a case report and review of the literature. Allergy Asthma Clin Immunol. (2022) 18(1):111. 10.1186/s13223-022-00749-0 36566211 PMC9789581

[42] Zhang Q Davis JC Lamborn IT Freeman AF Jing H Favreau AJ Combined immunodeficiency associated with DOCK8 mutations. N Engl J Med. (2009) 361(21):2046–55. 10.1056/NEJMoa0905506 19776401 PMC2965730

[43] Engelhardt KR McGhee S Winkler S Sassi A Woellner C Lopez-Herrera G Large deletions and point mutations involving the dedicator of cytokinesis 8 (DOCK8) in the autosomal-recessive form of hyper-IgE syndrome. J Allergy Clin Immunol. (2009) 124(6):1289–302.4. 10.1016/j.jaci.2009.10.038 20004785 PMC2818862

[44] Wilkie H Das M Pelovitz T Bainter W Woods B Alasharee M Regulatory T-cell dysfunction and cutaneous exposure to *Staphylococcus aureus* underlie eczema in DOCK8 deficiency. J Allergy Clin Immunol. (2024). 38185418 10.1016/j.jaci.2023.12.020

[45] Wilkie H Timilshina M Rahmayanti S Das M Pelovitz T Geha RS. DOCK8 is essential for neutrophil mediated clearance of cutaneous S. aureus infection. Clin Immunol. (2023) 254:109681. 10.1016/j.clim.2023.109681 37385324 PMC10529992

[46] Keles S Charbonnier LM Kabaleeswaran V Reisli I Genel F Gulez N Dedicator of cytokinesis 8 regulates signal transducer and activator of transcription 3 activation and promotes T(H)17 cell differentiation. J Allergy Clin Immunol. (2016) 138(5):1384–94.2. 10.1016/j.jaci.2016.04.023 27350570 PMC5099100

[47] Holland SM DeLeo FR Elloumi HZ Hsu AP Uzel G Brodsky N STAT3 mutations in the hyper-IgE syndrome. N Engl J Med. (2007) 357(16):1608–19. 10.1056/NEJMoa073687 17881745

[48] Farmand S Sundin M. Hyper-IgE syndromes: recent advances in pathogenesis, diagnostics and clinical care. Curr Opin Hematol. (2015) 22(1):12–22. 10.1097/MOH.0000000000000104 25469836

[49] Milner JD Brenchley JM Laurence A Freeman AF Hill BJ Elias KM Impaired T(H)17 cell differentiation in subjects with autosomal dominant hyper-IgE syndrome. Nature. (2008) 452(7188):773–6. 10.1038/nature06764 18337720 PMC2864108

[50] Puel A Cypowyj S Maródi L Abel L Picard C Casanova JL. Inborn errors of human IL-17 immunity underlie chronic mucocutaneous candidiasis. Curr Opin Allergy Clin Immunol. (2012) 12(6):616–22. 10.1097/ACI.0b013e328358cc0b 23026768 PMC3538358

[51] Minegishi Y Saito M Nagasawa M Takada H Hara T Tsuchiya S Molecular explanation for the contradiction between systemic Th17 defect and localized bacterial infection in hyper-IgE syndrome. J Exp Med. (2009) 206(6):1291–301. 10.1084/jem.20082767 19487419 PMC2715068

[52] Chandesris MO Melki I Natividad A Puel A Fieschi C Yun L Autosomal dominant STAT3 deficiency and hyper-IgE syndrome: molecular, cellular, and clinical features from a French national survey. Medicine (Baltimore). (2012) 91(4):e1–19. 10.1097/MD.0b013e31825f95b9 22751495 PMC3680355

[53] Farmand S Kremer B Häffner M Pütsep K Bergman P Sundin M Eosinophilia and reduced STAT3 signaling affect neutrophil cell death in autosomal-dominant hyper-IgE syndrome. Eur J Immunol. (2018) 48(12):1975–88. 10.1002/eji.201847650 30315710 PMC6587726

[54] Zhang Y Lin T Leung HM Zhang C Wilson-Mifsud B Feldman MB STAT3 mutation-associated airway epithelial defects in job syndrome. J Allergy Clin Immunol. (2023) 152(2):538–50. 10.1016/j.jaci.2022.12.821 36638921 PMC10330947

[55] Myles IA Anderson ED Earland NJ Zarember KA Sastalla I Williams KW TNF overproduction impairs epithelial staphylococcal response in hyper IgE syndrome. J Clin Invest. (2018) 128(8):3595–604. 10.1172/JCI121486 30035749 PMC6063472

[56] Béziat V Li J Lin JX Ma CS Li P Bousfiha A A recessive form of hyper-IgE syndrome by disruption of ZNF341-dependent STAT3 transcription and activity. Sci Immunol. (2018) 3(24). 10.1126/sciimmunol.aat4956 PMC6141026 29907691

[57] Spencer S Köstel Bal S Egner W Lango Allen H Raza SI Ma CA Loss of the interleukin-6 receptor causes immunodeficiency, atopy, and abnormal inflammatory responses. J Exp Med. (2019) 216(9):1986–98. 10.1084/jem.20190344 31235509 PMC6719421

[58] Béziat V Tavernier SJ Chen YH Ma CS Materna M Laurence A Dominant-negative mutations in human IL6ST underlie hyper-IgE syndrome. J Exp Med. (2020) 217(6). 10.1084/jem.20191804 PMC7971136 32207811

[59] Puel A Picard C Lorrot M Pons C Chrabieh M Lorenzo L Recurrent staphylococcal cellulitis and subcutaneous abscesses in a child with autoantibodies against IL-6. J Immunol. (2008) 180(1):647–54. 10.4049/jimmunol.180.1.647 18097067

[60] Lyons JJ Liu Y Ma CA Yu X O’Connell MP Lawrence MG ERBIN deficiency links STAT3 and TGF-β pathway defects with atopy in humans. J Exp Med. (2017) 214(3):669–80. 10.1084/jem.20161435 28126831 PMC5339676

[61] Toubiana J Okada S Hiller J Oleastro M Lagos Gomez M Aldave Becerra JC Heterozygous STAT1 gain-of-function mutations underlie an unexpectedly broad clinical phenotype. Blood. (2016) 127(25):3154–64. 10.1182/blood-2015-11-679902 27114460 PMC4920021

[62] Liu L Okada S Kong XF Kreins AY Cypowyj S Abhyankar A Gain-of-function human STAT1 mutations impair IL-17 immunity and underlie chronic mucocutaneous candidiasis. J Exp Med. (2011) 208(8):1635–48. 10.1084/jem.20110958 21727188 PMC3149226

[63] Picard C von Bernuth H Ghandil P Chrabieh M Levy O Arkwright PD Clinical features and outcome of patients with IRAK-4 and MyD88 deficiency. Medicine (Baltimore). (2010) 89(6):403–25. 10.1097/MD.0b013e3181fd8ec3 21057262 PMC3103888

[64] Picard C Casanova JL Puel A. Infectious diseases in patients with IRAK-4, MyD88, NEMO, or IκBα deficiency. Clin Microbiol Rev. (2011) 24(3):490–7. 10.1128/CMR.00001-11 21734245 PMC3131061

[65] Blanchard AC Waters VJ. Microbiology of cystic fibrosis airway disease. Semin Respir Crit Care Med. (2019) 40(6):727–36. 10.1055/s-0039-1698464 31887768 PMC7117079

[66] Dde C F Silva GR Cavalcante FS Carmo FL Fernandes LA Moreira S Methicillin-resistant *Staphylococcus aureus* in HIV patients: risk factors associated with colonization and/or infection and methods for characterization of isolates—a systematic review. Clinics (Sao Paulo). (2014) 69(11):770–6. 10.6061/clinics/2014(11)11 25518036 PMC4256048

[67] Crum-Cianflone NF Burgi AA Hale BR. Increasing rates of community-acquired methicillin-resistant *Staphylococcus aureus* infections among HIV-infected persons. Int J STD AIDS. (2007) 18(8):521–6. 10.1258/095646207781439702 17686212

[68] Shahrokh S Tabatabaee A Yazdi M Siavash M. Proportion of toxin and non-toxin virulence factors of *Staphylococcus aureus* isolates from diabetic foot infection: a systematic review and meta-analysis. BMC Microbiol. (2024) 24(1):1. 10.1186/s12866-023-03142-y 38172669 PMC10763345

[69] Tsai CM Caldera JR Hajam IA Chiang AWT Tsai CH Li H Non-protective immune imprint underlies failure of *Staphylococcus aureus* IsdB vaccine. Cell Host Microbe. (2022) 30(8):1163–72.6. 10.1016/j.chom.2022.06.006 35803276 PMC9378590

[70] Hassanzadeh H Baber J Begier E Noriega DC Konishi H Yato Y Efficacy of a 4-antigen *Staphylococcus aureus* vaccine in spinal surgery: the *STaphylococcus aureus* suRgical inpatient vaccine efficacy (STRIVE) randomized clinical trial. Clin Infect Dis. (2023) 77(2):312–20. 10.1093/cid/ciad218 37125490 PMC10371312

[71] Yanagisawa C Hanaki H Natae T Sunakawa K. Neutralization of staphylococcal exotoxins in vitro by human-origin intravenous immunoglobulin. J Infect Chemother. (2007) 13(6):368–72. 10.1007/s10156-007-0551-6 18095084

[72] Ishigame H Kakuta S Nagai T Kadoki M Nambu A Komiyama Y Differential roles of interleukin-17A and -17F in host defense against mucoepithelial bacterial infection and allergic responses. Immunity. (2009) 30(1):108–19. 10.1016/j.immuni.2008.11.009 19144317

[73] Cho JS Pietras EM Garcia NC Ramos RI Farzam DM Monroe HR IL-17 is essential for host defense against cutaneous *Staphylococcus aureus* infection in mice. J Clin Invest. (2010) 120(5):1762–73. 10.1172/JCI40891 20364087 PMC2860944

[74] Lévy R Okada S Béziat V Moriya K Liu C Chai LY Genetic, immunological, and clinical features of patients with bacterial and fungal infections due to inherited IL-17RA deficiency. Proc Natl Acad Sci U S A. (2016) 113(51):E8277–85. 10.1073/pnas.1618300114 27930337 PMC5187691

[75] Puel A Cypowyj S Bustamante J Wright JF Liu L Lim HK Chronic mucocutaneous candidiasis in humans with inborn errors of interleukin-17 immunity. Science. (2011) 332(6025):65–8. 10.1126/science.1200439 21350122 PMC3070042

[76] Schwerd T Twigg SRF Aschenbrenner D Manrique S Miller KA Taylor IB A biallelic mutation in IL6ST encoding the GP130 co-receptor causes immunodeficiency and craniosynostosis. J Exp Med. (2017) 214(9):2547–62. 10.1084/jem.20161810 28747427 PMC5584118

[77] de Beaucoudrey L Puel A Oe F-S Al C Ghandil P Chrabieh M Mutations in STAT3 and IL12RB1 impair the development of human IL-17–producing T cells. J Exp Med. (2008) 205(7):1543–50. 10.1084/jem.20080321 18591412 PMC2442631

[78] Corvilain E Casanova JL Puel A. Inherited CARD9 deficiency: invasive disease caused by ascomycete fungi in previously healthy children and adults. J Clin Immunol. (2018) 38(6):656–93. 10.1007/s10875-018-0539-2 30136218 PMC6157734

[79] Hsu AP Sowerwine KJ Lawrence MG Davis J Henderson CJ Zarember KA Intermediate phenotypes in patients with autosomal dominant hyper-IgE syndrome caused by somatic mosaicism. J Allergy Clin Immunol. (2013) 131(6):1586–93. 10.1016/j.jaci.2013.02.038 23623265 PMC4103905

[80] Bucciol G Moens L Bosch B Bossuyt X Casanova JL Puel A Lessons learned from the study of human inborn errors of innate immunity. J Allergy Clin Immunol. (2019) 143(2):507–27. 10.1016/j.jaci.2018.07.013 30075154 PMC6358521

[81] Rose-John S Winthrop K Calabrese L. The role of IL-6 in host defence against infections: immunobiology and clinical implications. Nat Rev Rheumatol. (2017) 13(7):399–409. 10.1038/nrrheum.2017.83 28615731

[82] Puel A Casanova JL. The nature of human IL-6. J Exp Med. (2019) 216(9):1969–71. 10.1084/jem.20191002 31235508 PMC6719420

[83] Ram S Lewis LA Rice PA. Infections of people with complement deficiencies and patients who have undergone splenectomy. Clin Microbiol Rev. (2010) 23(4):740–80. 10.1128/CMR.00048-09 20930072 PMC2952982

[84] Abu-Humaidan AH Elven M Sonesson A Garred P Sorensen OE. Persistent intracellular *Staphylococcus aureus* in keratinocytes lead to activation of the complement system with subsequent reduction in the intracellular bacterial load. Front Immunol. (2018) 9:396. 10.3389/fimmu.2018.00396 29545804 PMC5837974

[85] Spaan AN Neehus AL Laplantine E Staels F Ogishi M Seeleuthner Y Human OTULIN haploinsufficiency impairs cell-intrinsic immunity to staphylococcal α-toxin. Science. (2022) 376(6599):eabm6380. 10.1126/science.abm6380 35587511 PMC9233084

[86] Minegishi Y Saito M Morio T Watanabe K Agematsu K Tsuchiya S Human tyrosine kinase 2 deficiency reveals its requisite roles in multiple cytokine signals involved in innate and acquired immunity. Immunity. (2006) 25(5):745–55. 10.1016/j.immuni.2006.09.009 17088085

[87] Kreins AY Ciancanelli MJ Okada S Kong XF Ramírez-Alejo N Kilic SS Human TYK2 deficiency: mycobacterial and viral infections without hyper-IgE syndrome. J Exp Med. (2015) 212(10):1641–62. 10.1084/jem.20140280 26304966 PMC4577846

[88] Tuchscherr L Löffler B Proctor RA. Persistence of *Staphylococcus aureus*: multiple metabolic pathways impact the expression of virulence factors in small-colony variants (SCVs). Front Microbiol. (2020) 11:1028. 10.3389/fmicb.2020.01028 32508801 PMC7253646

