# Supplementary material for: Inborn errors of immunity with susceptibility to S. aureus infections
Source: Front Pediatr. 2024 Apr 24;12:1389650. doi: 10.3389/fped.2024.1389650 (PMC11078099; doi:10.3389/fped.2024.1389650)
Supplement: Supplementary file 1 [file Table1.pdf]

Disease	Genetic defect and inheritance (Examples)	Type of *S. aureus* infection and severity	Supposed mechanism associated to *S. aureus* susceptibility	Other infections	Additional clinical features	Typical laboratory features	Ref.
**IEI with neutropenia or deficient neutrophil functions leading to *S. aureus* disease**							
**Severe congenital neutropenia (SCN)**	- Neutrophil elastase defects (*ELANE*)<br>- HAX1 deficiency<br>- X-linked neutropenia (*WAS*)<br>- Shwachman-Diamond Syndrome (*SBDS, DNAJC21*)<br>- *G6PC3 def*<br>- *JAGN1 def*<br>- Barth Syndrome/3-Methylglutaconic aciduria type II (*TAZ*)<br>- Cohen syndrome (*VPS13B*)<br>- SMARCD2 def *(SMARCD2)*<br>- Specific granule deficiency (*CEBPE*)<br>- CXCR2 def (*CXCR2)*<br>*etc.*	Invasive infections<br>Blood stream infection<br>Abscesses<br>Recurrent skin infections<br><br>Severe infection with rapid clinical deterioration if not treated	Lack of neutrophils<br><br>Lack of adequate neutrophil release even during infections<br><br>SMARCD2: Myeloid differentiation defects, defect of granulopoiesis and neutrophil granule scarcity<br><br>Additional defects in neutrophil functions/bacterial killing e.g. specific granule deficiency due to CEBPE	Fungal infections e.g. *Candida*, *Aspergillus*<br><br>Severe infections due to gram negative bacteria<br><br>Fatal pyogenic infections with Pseudomonas<br><br>CMC but also invasive fungal infections possible	Gingivitis, oral ulcers<br><br>Depending on the underlying gene defect: e.g. cardiac defects in G6PC3 deficiency; Different morphological abnormalities in JAGN1 deficiency; Intermittent neutropenia, growth failure and exocrine pancreas insufficiency in Shwachman-Diamond-Syndrome; Developmental delay, skeletal anomalies, dysmorphic features, progressive myelofibrosis and MDS, chronic diarrhea in SMARCD2 def<br><br>Mutations in *ELANE* are responsible for around 50% of congenital neutropenia cases	Absent or very low neutrophils<br><br>Low IgG may be present in some SCN (e.g. JAGN1)<br><br>Some SCN may show specific finding in regards to neutrophil morphology (e.g. specific granule deficiency, SMARCD2 def, etc.)	(1)<br>(2)<br>(3)<br>(4)
**Autoimmune neutropenia (AIN)**	No specific gene, most frequently "benign" in infancy but may also occur in the wake of other IEI with autoimmune features particular if manifestation > 5 years of age	Skin infections, rarely abscesses<br><br>Tend to be less severe compared to SCN	Lack of neutrophils due to presence of autoantibodies<br><br>During infections increased output of neutrophils may overcome the quantitative defect	Mild CMC possible<br>Recurrent respiratory infections<br><br>Usually no severe fungal infections, no *Aspergillus spp.* or invasive infections	Oral ulcers<br>If benign AIN in infancy: usually otherwise healthy infant<br><br>In older children depending on the underlying cause, e.g. presence of other autoimmune conditions	Absent or very low neutrophils<br><br>Neutrophils may be normal during infections	(5, 6)
**Chronic granulomatous disease (CGD**)	XL CGD (XL *CYBB*): most common in non-consanguineous families, often more severe phenotype (early onset)<br><br>AR CGD (e.g. *CYBA, NCF1, NCF2, NCF4*)	Early onset recurrent and severe infections with *S. aureus* mostly affecting barriers: pneumonia, cutaneous abscesses, impetigo, granuloma formation, lymphadenitis<br><br>Through spread of pathogens: liver abscesses, osteomyelitis	Impaired phagocytic function and pathogen killing due to genetic defects in the ROS-producing NADPH oxidase complex	Recurrent and/or severe bacterial and fungal infections. Most common pathogens are catalase-positive bacteria (*S. aureus, Burkholderia, Serratia marcescens, Nocardia* spp.), mycobacteria, fungi (in particular *Aspergillus* spp.), and other opportunistic pathogens. Enteric gram-negative bacteria (*Escherichia coli* species, *Shigella* species, *Salmonella* species)	Hyperinflammation and autoimmunity (e.g. RA)<br><br>Inflammatory bowel disease that resembles Crohn's disease<br><br>Granuloma: key features of CGD and might cause symptoms of obstruction mainly in the gastrointestinal and urinary tract (associated weight loss, dysphagia etc.)<br><br>Delayed clinical presentation in case of residual function possible	Normal neutrophil numbers<br><br>Deficient release of ROS upon stimulation with PMA or *E. coli*<br><br>Anemia (due to chronic infection/inflammation)<br><br>Hypergammaglobulinemia (as a compensatory mechanism/sign of chronic immune stimulation)	(7)<br>(8)<br>(9)<br>(10)

Disease	Genetic defect and inheritance (Examples)	Type of *S. aureus* infection and severity	Supposed mechanism associated to *S. aureus* susceptibility	Other infections	Additional clinical features	Typical laboratory features	Ref.
**LAD**	AR LAD I (*ITGB2*), most frequent<br>AR LAD II (*SLC35C1*)<br>AR LAD III (*FERMT3*)<br><br>**LAD I**: mutation in ß2 integrin (CD18) (needed for firm adhesion of leukocytes)<br><br>**LAD II:** absence of the selectin fucosylated ligands (essential for initiating the rolling phase)<br><br>**LAD III:** mutations in kindlin-3 (key component for integrin activation)	Primarily infections of the skin and soft tissue, respiratory tract<br><br>Secondary bacteremia	Impaired adhesion and chemotaxis/phagocytosis leading to lack of neutrophils at the site of infection	**LAD I:** recurrent bacterial and fungal infections, primarily localized to skin and mucosal surfaces (otitis, cellulitis, pneumonia, respiratory tract infections), secondary bacteremia<br><br>**LAD II**: recurrent infections, less severe compared to LAD I and III<br><br>**LAD III**: (severe) recurrent infections	**LAD I**: omphalitis and delayed separation of umbilical cord<br><br>Delayed wound healing, oral ulcers, severe gingivitis, inflammatory bowel disease<br><br>**LAD II**: psychomotoric and mental retardation, short stature and facial stigmata, Bombay blood group due to absence of H-ag, periodontitis<br><br>**LAD III:** bleeding tendency, osteopetrosis-like bone defects. PJP described	Substantial leukocytosis with neutrophilia, but no pus at the site of infection	(11)<br>(12)<br>(13)
**Chediak-Higashi syndrome**	AR *(LYST)*<br><br>Also classified as SCN	*S. aureus* skin infections<br><br>Invasive infections particular if marked neutropenia	Impaired neutrophil chemotaxis, deficient intracellular bactericidal activity and deficient neutrophil degranulation due to granule disorders<br><br>Neutropenia	Recurrent pyogenic, bacterial infections of skin, mucous membranes and respiratory tract<br><br>Common pathogens apart from *S. aureus* are *S. pyogenes* and *S. pneumoniae*	Increased risk for hemophagocytic lymphohistiocytosis (HLH), dental-craniofacial changes, (peri)oral lesions (gingivitis). Partial oculocutaneous albinism, prolonged bleeding due to coagulation defects, immune and neurologic dysfunction such as developmental retardation, ataxia, tremor	Peripheral blood and bone marrow: presence of giant secretory granules in granulocytes<br><br>Neutropenia<br><br>Decreased NK and CTL degranulation	(14)<br>(15)
**G6PD deficiency class I**	XL G6PD def (*G6PD)*<br><br>**Note**: enhanced infection susceptibility only in very severe loss of enzyme activity (very rare)	unclear	G6PD supplies cells with NADPH, crucial for countering oxidative stress especially in red blood cells. Severe loss of enzyme activity may lead to NADPH oxidase deficiency in phagocytes<br>=> impaired phagocyte microbicidal activity due to low ROS generation	Pneumonia, arthritis, sepsis caused by similar pathogens as in CGD: *Salmonella* species, *Campylobacter jejuni*, *Pseudomonas aeruginosa*, *Staphylococcus epidermidis*, *Streptococci*, and *Aspergillus* species, Mycobacteria	Newborn jaundice<br><br>In case of acute hemolytic anemia: jaundice, pain and fatigue, renal damage	Acute hemolytic anemia due to risk factors of oxidative stress (e.g. medication, specific food) => anemia, hemoglobinuria	(16)<br>(17)

Disease	Genetic defect and inheritance (Examples)	Type of *S. aureus* infection and severity	Supposed mechanism associated to *S. aureus* susceptibility	Other infections	Additional clinical features	Typical laboratory features	Ref.
**Combined IEI with associated neutropenia (e.g. CD40L or CD40 deficiency)**	XL **CD40L** (*CD40LG*): more common<br><br>AR **CD40** deficiency (*CD40*)	Skin abscess/infections<br><br>Respiratory tract infection	Neutropenia and functional neutrophil defects with reduced ROS production<br><br>Bone marrow examinations may show myeloid maturation arrest at the pro-myelocyte stage with nearly complete lack of mature neutrophils	Impaired B-T-cell interaction and lack of class switch => combined IEI<br><br>Infections of the lungs and GI tract, e.g. pneumonia and diarrhea. Common pathogens are *Candida, P. aeruginosa*, *Klebsiella* spp.<br><br>Opportunistic infections by *Aspergillus* spp., *Pneumocystis jirovecii* (PJP), *Toxoplasma gondii*, *Cryptosporidium* spp. and *Cryptococcus* spp.	Autoimmune diseases, sclerosing cholangitis (CD40>CD40L), hepatitis, malignancies (liver, biliary tract), oral ulcers, proctitis, impairment of height and weight, chronic/recurrent diarrhea	**CD40L** def and **CD40** def:<br>Low IgG, IgE, IgA<br>Normal or elevated IgM<br><br>Neutropenia<br><br>**CD40:** impaired memory B-cell generation	(18)<br>(19)<br>(20)
**Neutropenia or Neutrophil dysfunction related to RAC2**	Different mutations leading to variable phenotype:<br><br>AD **RAC2-LOF**:<br>LAD-like disease<br><br>AD **RAC2 GOF**:<br>(S)CID phenotype<br><br>AR **RAC2-Deficiency**: CVID phenotype	**RAC2 LOF:** abscesses<br><br>**RAC2 GOF:** pulmonary infections, cellulitis	RAC2 is an essential regulator of neutrophil chemotaxis. RAC2 is involved in NADPH oxidase function to certain stimuli.<br>**RAC2 LOF:** impaired bacterial killing, chemotaxis defect.<br>**RAC2 GOF:** neutropenia	**RAC2 GOF**: recurrent bacterial (*Mycobacterium* spp.), fungal (*Aspergillus* spp.) and viral infections (HSV), pneumonia, sepsis, urinary tract infections, recurrent sinusitis	**RAC2 LOF:** defective wound healing, lack of pus at infection site<br><br>**RAC2 GOF:** lymphoproliferation	**RAC2 LOF**: leukocytosis and neutrophilia. Defective neutrophil chemotaxis and ROS release (fMLP induced, but normal to PMA).<br>**RAC2 GOF**: low T cells, B cells, and NK cells, myeloid dysfunction, low IgG with impaired specific antibody responses, deficient neutrophil chemotaxis, enhanced ROS	(21)<br>(22)<br>(23)<br>(24)<br>(25)
**Combined IEI with syndromic features and with associated neutropenia (e.g. PGM3 deficiency)**	AR PGM3 def (*PGM3*)	Recurrent skin abscesses, pneumonia	Neutropenia<br><br>PGM3 deficiency is a congenital glycosylation defect. Impaired glycosylation of immune receptors, immunoglobulins, etc. => deficient immune response	*Candida* infections<br>Viral infections (VZV, RSV)<br><br>Recurrent upper/lower respiratory tract infections, otitis and bronchiectasis	Widespread clinical manifestations: connective tissue and skeletal abnormalities (e.g. short statue, hyperextensibility, brachydactyly, scoliosis, facial stigmata), severe atopy/autoimmunity, developmental delay and psychomotoric disorders	IgE is often markedly elevated<br>Variable eosinophilia<br><br>Low CD4+ and CD8+ T cells<br>Borderline low Th17 cells<br>Low B and B memory cells<br><br>Neutropenia	(26)<br>(27)<br>(28)
**Combined IEI with severe eczema/atopic dermatitis (e.g. DOCK8 deficiency)**	AR DOCK8 deficiency	Skin infections/ dermatitis/folliculitis<br><br>Less frequent invasive infections (e.g. osteomyelitis)	Severe atopic skin changes with enhanced *S. aureus* colonization but also deterioration of eczema by *S. aureus.*<br><br>Enhanced neutrophil death by *S. aureus (*murine data). Reduced STAT3 phosphorylation	Combined immunodeficiency with reduced lymphocyte function and lymphopenia<br><br>Severe cutaneous viral infections: HSV/VZV/Molluscum<br><br>Mycobacterial infections	Severe atopic disease/allergies<br><br>Cancer predisposition	Eosinophilia<br>Markedly elevated IgE<br>Low Th17 cells<br>Deficient lymphocyte proliferation, lymphopenia, reduced memory B-/T-cells, low naïve CD8, Treg dysfunction, low IgM, normal/high IgG, reduced specific antibody responses	(29-32)

Disease	Genetic defect and inheritance (Examples)	Type of *S. aureus* infection and severity	Supposed mechanism associated to *S. aureus* susceptibility	Other infections	Additional clinical features	Typical laboratory features	Ref.
**IEI with defective cytokine signaling leading to enhanced *S. aureus* susceptibility**							
**STAT3-deficient HIES**	AD LOF STAT3 (*STAT3*)<br><br>Residual STAT3 signaling is present	Skin infections,<br>(cold) abscesses,<br>pneumonia<br><br>Severe infections with extended tissue damage but discrepancy in regards to clinical symptoms e.g. lack of fever and CRP elevation, no typical signs of inflammation due to altered cytokine signaling	Multiple cytokines signal via STAT3, including in particular IL-6 and IL-10, as well as IL-11, IL-21, IL-23 and others. Impaired IL-6 signaling has been reported to be associated with recurrent staphylococcal infections<br><br>Potential contribution of reduced Th17 cells and associated reduction of antimicrobial peptides (e.g. Reg3γ, CXCL8, ß-defensin) on epithelial surface<br><br>Reduced transepithelial resistance to injury through decreased IL-22 secretion as well as epithelial dysfunction through TNF-α overproduction and disordered EMT<br><br>Enhanced neutrophil cell death upon contact with *S. aureus*	*Candida* (CMC)<br><br>Pneumonia due to *S. pneumonia, H. influenzae*, nontuberculous *Mycobacteria*<br><br>Opportunistic fungal infection of damaged lung tissue: Pulmonary infections due to *Pneumocystis jiroveci, P. aeruginosa,* or *Aspergillus spp.* might also occur as secondary opportunistic infections in damaged lungs<br><br>Viral reactivation, especially VZV	Facial dysmorphism and retained primary teeth<br><br>Pneumatocele and bronchiectasis<br><br>Eczema, connective tissue, vascular, and skeletal abnormalities<br><br>GI symptoms such as gastroesophageal reflux, eosinophilic gastritis<br><br>Hodgkin and non-Hodgkin-Lymphoma<br><br>Fever may be absent or low even during severe infections	Eosinophilia<br>Markedly elevated IgE<br><br>Specific antibody production partly reduced due to class switch failure although IgG levels are normal (B-cell differentiation problem)<br><br>Diminished memory B and T cells<br><br>Low Th17 cells<br><br>Low central memory CD4 and CD8 T-cells<br><br>CRP may be low even during severe infections	(33)<br>(34)<br>(35)<br>(36)<br>(37)<br>(38)<br>(39)
**ZNF341 deficiency**	AR *ZNF341*	Skin and soft tissue infections including (cold) abscesses, osteomyelitis, pneumonia	ZNF341 is a transcription factor. It interacts with the STAT3 gene promoter region. Thus, ZNF341 deficiency leads to impaired STAT3 signaling, and its phenotype resembles HIES-STAT3 deficiency<br><br>As in STAT3-deficient patients, impaired STAT3/IL6-interaction might explain susceptibility to *S. aureus*	Recurrent *Candida* spp. infections, recurrent respiratory infections	Resembles STAT3-deficient HIES: Atopy, eczema<br>Skeletal abnormalities with facial dysmorphism, retained primary teeth, formation of bronchiectasis and pneumatoceles<br><br>Patients are reported to display more inflammation as STAT3-deficient HIES patients, e.g. CRP and fever may be present in some	Elevated IgE, Eosinophilia<br>Reduced memory B cells<br>Partly deficient specific antibody responses<br><br>IgG may be elevated<br><br>Decreased Th17 and NK cells<br><br>Low central memory CD4 and CD8 T-cells	(40)<br>(41)

Disease	Genetic defect and inheritance (Examples)	Type of *S. aureus* infection and severity	Supposed mechanism associated to *S. aureus* susceptibility	Other infections	Additional clinical features	Typical laboratory features	Ref.
**IL6 signal transducer deficiency (IL6ST)**	Partial LOF due to AR IL6ST def (LOF *IL6ST*)<br>AD IL6ST def<br>(DN *IL6ST*)<br><br>IL6ST encodes GP130, a receptor subunit used by the IL-6 cytokine family (e.g. IL-6, IL-11, IL-27, LIF, etc...)<br><br>**Note:** Complete IL6ST LOF is perinatally lethal	**Partial LOF IL6ST**: Recurrent and/or severe lung infections (pneumonia)<br><br>**DN IL6ST**: (cold) skin abscesses and severe pneumonia, osteomyelitis	Impaired IL-6 signaling	**Partial LOF IL6ST**: Severe and recurrent respiratory tract infections, soft tissue infections caused by *Streptococcus spp*.<br><br>**DN IL6ST:**<br>CMC in some patients, infections with *Aspergillus spp*. Lung infections caused *by H. influenzae*<br><br>Secondary lung damage with bronchiectasis	**Partial LOF and DN IL6ST:** Eczema, asthma, retention of primary teeth, scoliosis<br><br>More frequent in **partial LOF IL6ST**: craniosynostosis, Reduced/lacking inflammatory response during infections<br><br>**DN IL6ST:** Fever and CRP elevation may be present (depending on the degree of abolished signaling). Pneumotocele reported	**Partial LOF IL6ST:**<br>Elevated IgE<br>Eosinophilia<br>Decreased memory B cells<br>Low to normal Th17 cells<br>Impaired acute-phase-response despite severe infections<br>**DN IL6ST:**<br>Elevated IgE<br>Eosinophilia<br>Reduced memory B cells<br>BUT normal Th17 numbers	(42)<br>(43)<br>(44)<br>(45)
**IL6 receptor deficiency**	AR IL6R def (*IL6R*)	Skin and soft tissue ("cold") abscesses, pneumonia	Impaired IL-6 signaling	Sinopulmonary infections caused by other pathogens such as *H. influenzae*<br><br>No fungal infections described	Allergic/Atopic diseases: Atopic dermatitis, asthma<br><br>Clinically inadequate response to infections, "cold" abscesses, absent or low-grade fever during infections	Eosinophilia, elevated IgE<br>Elevated IL6<br>No CRP during infection<br>Th17 cells are usually normal<br>Reduced classed switched memory B cells<br>Low IgG, IgA, IgM	(46)
**Tyk2 deficiency**	AR TYK2 (*TYK2*)<br><br>Different mutations of Tyk2 have been identified with different phenotypes	Only patient 1 (P1)*:*<br>*S aureus* abscesses<br><br>Patients 2-25: no *S. aureus* infections!	**P1-P8:** impaired IL-10, IL-12, IL-23, and IFN-α/β responses. Insufficient Inf-γ induction. **P1:** in addition, impaired IL-6 signaling, possibly accounting for the *observed S. aureus* susceptibility. **Deficient IL-6 signaling unrelated to Tyk2 def**	Recurrent intracellular bacterial infections, mycobacteria and viral infections	**P1:** Atopic dermatitis	**P1**: Hyper-IgE phenotype with Eosinophilia and elevated IgE	(47)<br>(48)<br>(49)<br>(50)
**ERBIN deficiency**	AD *ERBB2IP*<br>ERBB2-interacting protein (ERBIN)<br>ERBIN forms a complex with STAT3 and SMAD2/3 which has an inhibitory effect on TGF-β. Unregulated TGF-β leads to increased Th2 cytokines, Treg and IL-4Rα expression	Susceptibility to *S. aureus* mentioned but not specified	ERBIN def shows overlapping features with STAT3-def HIES, however the precise contribution to *S. aureus* defense and exact pathomechanism for infection susceptibility is currently unknown	Respiratory tract infections mentioned, not specified<br><br>No recurrent fungal infections described	Eczema, eosinophilia, connective tissue abnormalities, skeletal and vascular abnormalities	Mildly elevated IgE<br>Mild Eosinophilia<br><br><br>Normal memory B cell<br>Normal specific IgG<br>Normal Th17 cell numbers	(51)

Disease	Genetic defect and inheritance (Examples)	Type of *S. aureus* infection and severity	Supposed mechanism associated to *S. aureus* susceptibility	Other infections	Additional clinical features	Typical laboratory features	Ref.
**STAT1GOF**	AD STAT1 GOF	Mostly skin (folliculitis, cellulitis, abscesses) and less frequently respiratory tract infections	Not precisely known, potential explanation: breaches in skin by severe candida infections together with reduced Th17 cells affecting *S. aureus* defense at the skin and mucosa sites<br><br>Potentially also antagonization of STAT3 response to IL-6 by enhanced STAT1 activation	Most prominent CMC<br>Invasive fungal infection due to *Candida, Aspergillus, Cryptococcus* possible<br>PJP pneumonia reported<br>Bacterial infections also due to *Streptococcus* spp., *Pseudomonas* and *H. influenzae*<br><br>Skin infection with Herpesviridae<br>Mycobacterial disease	Autoimmunity (Hypothyroidism, cytopenia, SLE)<br><br>Cerebral aneurysm<br><br>Cancer (squamous cell and GI carcinoma)<br><br>Bronchiectasis, dysphagia, failure to thrive	Low Th17 cells<br><br>Variable findings regarding B-cell immunity: Lack of specific antibodies, IgG-subclass deficiency and low Memory B-cells possible. IgE usually normal.<br><br>Enhanced STAT1 phosphorylation	(52)<br>(53)<br>(54)
**Defects in TLR and/or IL1R signaling with enhanced susceptibility to *S. aureus***							
**TLR signaling deficiencies**	IRAK-4 def (AR *IRAK4)*<br>MyD88 def (AR *MyD88)*<br><br>XR-EDA-ID due to NEMO/IKBKG def (XL *IKBKG)*<br>AD-EDA-ID due to IKBA GOF mutation (AD *NFKBIA)* leading to impaired IκBα degradation<br><br>TIRAP def (AR *TIRAP, LOF)*	**IRAK-4** and **MyD88** def: invasive pyogenic infections (meningitis, sepsis, arthritis, osteomyelitis, abscesses), caused by *S. aureus* (and particularly caused by *S. pneumoniae).* Noninvasive infections (skin URT, ENT) caused by *S. aureus*<br><br>**NEMO def** and **IKBA GOF:** invasive pyogenic infections with *S. aureus*<br><br>**TIRAP def:** Severe invasive *staphylococcal* infection in 1 patient (pneumonia, sepsis) with concomitantly absent LTA-antibodies	**IRAK-4** and **MyD88** def: Impaired antigen recognition and response through defects in TLR-signaling and IL-1R-signalling. Impaired cytokine production (IL-6, IL-8) with impaired ability to increase CRP and mount fever; staphylococcal infections may result from impaired IL6-signaling<br><br>**NEMO** and **IKBA GOF**: impaired TLR-signaling and impaired alternative TRIF-dependent signaling<br><br>**TIRAP def:** TIRAP acts as a bridging molecule for MyD88 in the context of TLR2 and TLR4 activation. Impaired responses to TLR2 and TLR4 stimulation, suggested only to be relevant in the absence of LTA-antibodies which enhance TLR2 response to staphylococcal LTA	Encapsulated bacteria<br><br>**IRAK-4** and **MyD88** deficiency: susceptibility to infections with *S. pneumoniae* and *P. aeruginosa*<br><br>**NEMO def** and **IKBA GOF**: broad susceptibility to infections with invasive pyogenic bacteria caused by *S. pneumoniae* or *H. influenzae*, *P. aeruginosa, Klebsiella pneumoniae, Serratia marcescens*, environmental mycobacteria, *Candida*, PJP, and, to a lesser extent, viruses (HSV, CMV more frequent in NEMO def)	**IRAK-4 def** and **MyD88 def**: pus formation but lack of fever<br><br>**IRAK-4 def**: delayed separation of the umbilical cord<br><br>**NEMO def** and **IKBA GOF**: anhidrotic ectodermal dysplasia (EDA) with sparse hair, abnormal teeth (conical teeth, tooth agenesis), and hypohidrosis, +/-colitis<br><br>In some NEMO-deficient patients, associated osteopetrosis and/or lymphedema has been described	**IRAK-4 def** and **MyD88 def**:<br>Lack of IL-6 production<br>Lack of CRP,<br>Impaired IgG and IgM to pneumococcus<br>Elevated IgE and IgG4<br><br>**NEMO-def and IKBA GOF:**<br>Lack of IL-6 and IL-10 production,<br>Impaired antibody response (protein and pneumococcus antibodies)<br>Hypogammaglobulinemia,<br>May have reduced memory CD4 and CD8 T cells	(55)<br>(56)<br>(57)<br>(58)

Disease	Genetic defect and inheritance (Examples)	Type of *S. aureus* infection and severity	Supposed mechanism associated to *S. aureus* susceptibility	Other infections	Additional clinical features	Typical laboratory features	Ref.
**Complement deficiencies with enhanced susceptibility to *S. aureus***							
**Complement-defects**	Various defects e.g. homozygous C2-deficiency	Severe infections with S. *aureus* have been described in complete C3 and C2 deficiency<br><br>The role of complement in the defense against *S. aureus* appears less pronounced compared to its critical function in combating other encapsulated bacteria	Impaired detection of microbial pathogens which leads to impaired lysis or opsonization and thus reduced microbial clearance	Depends on the protein:<br>C1r/s, C1q, C2, C3, C4: Infections with encapsulated bacteria (*H. influenzae, N. meningitidis, S. pneumoniae*): sepsis, pneumonia, meningitis<br><br>C5-C9: Meningitis, sepsis due to *N. meningitidis*<br><br>MBL-deficiency in patients with underlying condition may predispose to mostly respiratory tract or skin infections	Deficiency of C1r/s, C1q, C2 or C4: SLE or SLE-like disease<br><br>C3-deficiency: glomerulonephritis	Normal white blood cell counts<br>Normal IgG levels<br><br>Abnormal CH50 assay in deficiencies of classical pathway.<br>Abnormal AH50 assay in deficiencies of components of alternative pathway	(59)<br>(60)<br>(61)<br>(62)
**IEI affecting non-leukocytic cells leading to enhanced susceptibility to *S. aureus* infections**							
**Otulin haplo-insufficiency**	AD OTULIN	Life-threatening necrotizing infections of the skin, soft tissue and the lungs	Impaired innate immunity of non-leukocytic cells to staphylococcal α-toxin. OTULIN haploinsufficiency causes an accumulation of caveolin-1 which enhances cytotoxicity to α-toxin in dermal fibroblasts	Necrosis might appear without *S. aureus* being detectable, suggesting low-grade infectious or, non-infectious triggers	First episode of disease most frequently during adolescence	Accumulation of caveolin-1 in non-hematopoietic cells (caveolin-1 clusters with α-toxin → α-toxin induced cell death).	(63)
**Secondary immunodeficiencies with enhanced susceptibility to *S. aureus* infection**							
**HIV Infection**	Not applicable	Cutaneous or subcutaneous infections (psoas abscess), bacteremia	Chronic colonization (MRSA, nasal carriage), presence of vascular catheter, associated neutropenia (end-stage AIDS, secondary neutropenia due to therapy), the extent to which low CD4 counts contribute to risk of *S. aureus* infections remains unclear	Related to the activity of HIV infection:<br>opportunistic infections: Candidiasis, Herpes zoster, oral hairy leukoplakia<br><br>AIDS-defining infections (severe opportunistic infections, JC-Viral infection, TBC, PJP etc.)	Weight loss, fatigue, subfebrile temperature, chronic diarrhea, lymph node swelling, dysplasia and neoplasia, neuropathies, nephropathies<br><br>AIDS-defining wasting syndrome, AIDS-defining cancers (e.g. Kaposi-sarcoma), neurological dysfunction	Reduced CD4+ cells<br><br>HIV-associated neutropenia or thrombocytopenia<br><br>HIV antigen/antibody test positive<br><br>HIV PCR positive in blood	(64)<br>(65)<br>(66)
**Influenza Infection**	Not applicable	Secondary bacterial or *S. aureus* co-infection (e.g. pneumonia or blood stream infection) associated with complicated outcome	Several mechanisms suggested leading to altered cytokine signaling and blunted immune response<br>Enhanced risk in MRSA colonized patients or patients with recent *S. aureus* skin infection	Pneumonia due to *S. pneumoniae* also frequent during/after influenza	Frequently clinical deterioration after improvement or lack of improvement after 3-5 days<br><br>May affect otherwise healthy individuals	Lymphopenia, neutropenia<br><br>Detection of influenza virus	(67)<br>(68)

Disease	Genetic defect and inheritance (Examples)	Type of *S. aureus* infection and severity	Supposed mechanism associated to *S. aureus* susceptibility	Other infections	Additional clinical features	Typical laboratory features	Ref.
**Medications causing neutropenia**	Not applicable	Invasive infections: blood stream infection/sepsis<br>In particular if central lines are present	Neutropenia for example due to chemotherapy<br><br>Risk is also related to local epidemiology, presence of central lines and colonization	Severe and frequent infections due to gram negative bacteria (*E. coli*, *Klebsiella* spp., *Pseudomonas* spp.)<br><br>CMC but also invasive fungal infections (*Candida, Aspergillus*)	Depending on the cause for the medication, e.g. chemotherapy in patients with malignancies but could also affect otherwise healthy individuals e.g. in rare cases of medication-induced agranulocytosis	Absent or very low neutrophils	(69)<br>(70)<br>(71)
**Phenocopies of IEI: Autoantibodies against IL-6**	Not applicable	Skin and soft tissue infections (abscesses), 1 case with sepsis reported	Neutralizing auto-antibodies against IL-6, impaired IL6-signaling	*E. Coli, S. pneumoniae*	Fever may or may not be present	No CRP increase despite clinical signs of inflammation<br>Neutrophilia possible<br>Elevated PCT reported	(72)<br>(73)<br>(74)
**Medications affecting IL-6 signaling**	Not applicable	Skin/soft tissue/abscesses, Case reports (adults): Invasive infections (sepsis, meningitis, pneumonia, septic arthritis)	Reduced IL-6 signaling for example due to IL-6 receptor blockage	*Streptococcus spp.* Depending on underlying disease, age and co-medication	Fever may be absent in spite of severe systemic infection.<br>Patients with previous *S. aureus* infection may be at higher risk	No CRP increase despite clinical signs of inflammation<br>Neutrophilia<br>Variable PCT response	(75)<br>(76)<br>(77)
**Other diseases with enhanced susceptibility to *S. aureus* infections**							
**Cystic fibrosis (CF)**	AR CFTR	Recurrent pulmonary infections (leading to lung damage and bronchiectasis)<br><br>High rates of colonization: *S. aureus*: younger children<br>MRSA: young adults	Abnormal mucous production and clearance, which leads to chronic infection and unregulated inflammation<br><br>Observed adhesion defect in monocytes of patients with cystic fibrosis due to mutations in CFTR was termed LAD-IV. Contribution for the *S. aureus* phenotype not clear, yet	In small children *S. aureus* and *H. influenzae* are common, later on the most common bacteria is *Pseudomonas aeruginosa.* Other typical pathogens include *Burkholderia cepacia and Stenotrophomonas maltophilia*<br><br>*Aspergillus* and *Non-tuberculous mycobacteria*	Bronchiectasis<br>Digestive system disorders: pancreas insufficiency leading to fat malabsorption, meconium ileus, rectal prolapse, failure to thrive.<br>Diseases of liver and biliary system: cirrhosis, fibrosis<br><br>Skeletal involvement: clubbed fingers, scoliosis, reduced bone density with fractures	Positive sweat chloride test	(78)<br>(79)<br>(80)
**Diabetes mellitus**	Monogenetic diabetes (*HNF1a, GCK, HNF4*)<br>T1DM not applicable<br>T2DM not applicable	Infections of the skin and soft tissue, *S. aureus* is supposed to be the leading cause of diabetic food infection<br><br>Risk of *S. aureus* pneumonia	Impaired wound healing due to hyperglycemia and angiopathy<br><br>Impaired glucose metabolism associated with decreased ROS production and deficient cytokine signaling of various cytokines	Enhanced frequency of respiratory, gastrointestinal and genitourinary infections. *P. aeruginosa* (Otitis externa maligna), *Klebsiella pneumoniae, Candida*	**T1DM**: Polydipsia, polyuria, and weight loss, visual disturbances<br><br>**T2DM**: obesity, metabolic syndrome	Elevated plasma glucose<br>Pathological OGTT<br>HbA1c ≥6.5 percent<br>Ketonemia/Ketoazidosis<br>Glucosuria<br>Ketonuria	(81)<br>(82)<br>(83)<br>(84)<br>(85)
**Colonization with virulent *S. aureus* strain**	Virulence genes of the *S. aureus:* e.g. PVL	Depending on the virulence genes of the *S. aureus*: from recurrent superficial skin infections to invasive disease (e.g. pneumonia)	Depending on the virulence genes, e.g. presence of PVL: leucocidin activity => elevated neutrophil cell death	Not applicable	May affect otherwise healthy individuals	Normal laboratory findings in regards to known host risk factors<br>Detection of specific *S. aureus* strains or virulence genes upon microbiological testing	(86)<br>(87)

Suppl. Table 1: IEI, secondary immunodeficiencies and other diseases with enhanced susceptibility to *S. aureus*

IEI with susceptibility to *S. aureus* are grouped according to their most prevalent immunological dysfunction associated to *S. aureus*. Additional characteristic infections, clinical and typical laboratory features are displayed in order to facilitate detection of the underlying disease depending on the clinical presentation. Genetic causes are listed exemplary and do not claim to be complete. Secondary immunodeficiencies and other disease with susceptibility to *S. aureus* are also displayed. This table is meant as an overview, for details regarding single disease entities reading of primary literature is recommended.

Abbreviations: *AD* – autosomal-dominant, *AH50* - alternative hemolytic complement activity, *AIDS* - acquired immune deficiency syndrome, *AR* – autosomal-recessive, *C2*– complement component 2, *CD* - cluster of differentiation, *CD40* - cluster of differentiation 40, *CD40L* - CD40 ligand, *CD40LG* - CD40 ligand gene, *CEBPE* - CCAAT enhancer binding protein epsilon, *CF* - cystic fibrosis, *CGD* - chronic granulomatous disease, *CH50* - 50% hemolytic complement activity, *CMC* - chronic mucocutaneous candidiasis, *CRP* - c-reactive protein, *CTFR* - cystic fibrosis transmembrane conductance regulator, *CTL* – cytotoxic T lymphocyte, *CXCR2* - C-X-C motif chemokine receptor 2, *CYBA* - cytochrome B-245 alpha chain, *CYBB* - cytochrome B-245 beta chain, *CYBC1* - cytochrome B-245 chaperone 1, *DNAJC21* - DnaJ heat shock protein family (Hsp40) member C21, *Def* - deficiency, *DOCK8* - dedicator of cytokinesis 8, *E. Coli* - Escherichia Coli, *e.g.* – exempli gratia, for example, *ELANE* – elastase neutrophil expressed, *EMT* – epithelial-mesenchymal transition, *ERBIN* - ERBB2-interacting protein, *FERMT3* - fermitin family member 3, *G6PD* - glucose-6-phosphate dehydrogenase, *GOF* - gain of function, *HbA1c* - hemoglobin A1c, *HIES* - hyper IgE syndrome, *HIV* - human immunodeficiency virus, *HLH* - hemophagocytic lymphohistiocytosis, *IEI* - inborn errors of immunity, *IKBKG* - inhibitor of nuclear factor kappa B kinase regulatory subunit gamma, *IL* - interleukin, *IL6R* - interleukin 6 receptor, *IL6ST* - interleukin 6 signal transducer, *Ig* - immunoglobulins, *IRAK-4* - interleukin 1 receptor associated kinase 4, *ITGB2* - integrin subunit Beta 2, *LAD* - leukocyte adhesion deficiency, *LYST* - lysosomal trafficking regulator, *MRSA* - methicillin-resistant Staphylococcus aureus, *MyD88* - myeloid differentiation primary response 88, *NADPH* - nicotinamide adenine dinucleotide phosphate, *NCF* - neutrophil cytosolic factor, *NEMO* - NF-kappa B essential modulator, *NF-κB* - nuclear factor kappa B, *NFKBIA* - nuclear factor kappa B inhibitor alpha, *NK* – natural killer, *OGTT* - oral glucose tolerance test, *OTULIN* - OTU deubiquitinase with linear linkage specificity, *PCT* - procalcitonin, *PGM3* - phosphoglucomutase 3, *PJ(P)* - pneumocystis jirovecii (pneumonia), *PMA*- phorbol myristate acetate, *RA* – rheumatoid arthritis, *RAC2* - ras-related C3 botulinum toxin substrate 2, *ROS* - reactive oxygen species, *S. aureus* – Staphylococcus aureus, *SBDS* - Shwachman-Bodian-Diamond syndrome, *SCN* - severe congenital neutropenia, *SLC35C1* - solute carrier family 35 member C1, *SLE* - systemic lupus erythematosus, *SMARCD2* - SWI/SNF related, matrix associated, actin dependent regulator of chromatin, subfamily D, member 2, *S. pneumoniae* – Streptococcus pneumoniae, *S. pyogenes* – Streptococcus pyogenes, *Spp.* – species pluralis, *TBC* – tuberculosis, *TIRAP* - toll/interleukin 1 receptor (TIR) domain containing adaptor protein, *TLR* - toll-like receptor, *Treg cells* – T regulatory cells, *T1DM* – type 1 diabetes mellitus, *T2DM* – type 2 diabetes mellitus, *VZV* - varicella zoster virus, *WAS* - wiskott-aldrich syndrome, *XL* - X-linked, *ZNF341* - zinc finger protein 341

References

1. Skokowa J, Dale DC, Touw IP, Zeidler C, Welte K. Severe congenital neutropenias. Nat Rev Dis Primers. 2017;3:17032.
2. Han X, Lu S, Gu C, Bian Z, Xie X, Qiao X. Clinical features, epidemiology, and treatment of Shwachman-Diamond syndrome: a systematic review. BMC Pediatr. 2023;23(1):503.
3. Witzel M, Petersheim D, Fan Y, Bahrami E, Racek T, Rohlfs M, et al. Chromatin-remodeling factor SMARCD2 regulates transcriptional networks controlling differentiation of neutrophil granulocytes. Nat Genet. 2017;49(5):742-52.
4. Banday AZ, Kaur A, Akagi T, Bhattarai D, Muraoka M, Dev D, et al. A Novel CEBPE Variant Causes Severe Infections and Profound Neutropenia. J Clin Immunol. 2022;42(7):1434-50.
5. Bux J, Behrens G, Jaeger G, Welte K. Diagnosis and clinical course of autoimmune neutropenia in infancy: analysis of 240 cases. Blood. 1998;91(1):181-6.
6. Farruggia P, Dufour C. Diagnosis and management of primary autoimmune neutropenia in children: insights for clinicians. Ther Adv Hematol. 2015;6(1):15-24.
7. Buvelot H, Posfay-Barbe KM, Linder P, Schrenzel J, Krause K-H. Staphylococcus aureus, phagocyte NADPH oxidase and chronic granulomatous disease. FEMS Microbiology Reviews. 2016;41(2):139-57.
8. Roos D. Chronic granulomatous disease. Br Med Bull. 2016;118(1):50-63.
9. Justiz-Vaillant AA, Williams-Persad AF, Arozarena-Fundora R, Gopaul D, Soodeen S, Asin-Milan O, et al. Chronic Granulomatous Disease (CGD): Commonly Associated Pathogens, Diagnosis and Treatment. Microorganisms. 2023;11(9).
10. Meissner F, Seger RA, Moshous D, Fischer A, Reichenbach J, Zychlinsky A. Inflammasome activation in NADPH oxidase defective mononuclear phagocytes from patients with chronic granulomatous disease. Blood. 2010;116(9):1570-3.
11. Fekadu J, Modlich U, Bader P, Bakhtiar S. Understanding the Role of LFA-1 in Leukocyte Adhesion Deficiency Type I (LAD I): Moving towards Inflammation? Int J Mol Sci. 2022;23(7).
12. Almarza Novoa E, Kasbekar S, Thrasher AJ, Kohn DB, Sevilla J, Nguyen T, et al. Leukocyte adhesion deficiency-I: A comprehensive review of all published cases. J Allergy Clin Immunol Pract. 2018;6(4):1418-20.e10.
13. Niethammer D, Dieterle U, Kleihauer E, Wildfeuer A, Haferkamp O, Hitzig WH. An inherited defect in granulocyte function: impaired chemotaxis, phagocytosis and intracellular killing of microorganisms. Helv Paediatr Acta. 1976;30(6):537-41.
14. de Arruda JAA, Sousa-Neto SS, Abreu LG, Schuch LF, Souza VG, Alves TVL, et al. Oral manifestations of Chediak-Higashi syndrome: A systematic review. Dis Mon. 2023;69(1):101356.
15. Sharma P, Nicoli ER, Serra-Vinardell J, Morimoto M, Toro C, Malicdan MCV, Introne WJ. Chediak-Higashi syndrome: a review of the past, present, and future. Drug Discov Today Dis Models. 2020;31:31-6.
16. Siler U, Romao S, Tejera E, Pastukhov O, Kuzmenko E, Valencia RG, et al. Severe glucose-6-phosphate dehydrogenase deficiency leads to susceptibility to infection and absent NETosis. J Allergy Clin Immunol. 2017;139(1):212-9.e3.
17. Sun B, Li Q, Dong X, Hou J, Wang W, Ying W, et al. Severe G6PD deficiency leads to recurrent infections and defects in ROS production: Case report and literature review. Front Genet. 2022;13:1035673.
18. Cabral-Marques O, França TT, Al-Sbiei A, Schimke LF, Khan TA, Feriotti C, et al. CD40 ligand deficiency causes functional defects of peripheral neutrophils that are improved by exogenous IFN-γ. J Allergy Clin Immunol. 2018;142(5):1571-88.e9.
19. Banday AZ, Nisar R, Patra PK, Kaur A, Sadanand R, Chaudhry C, et al. Clinical and Immunological Features, Genetic Variants, and Outcomes of Patients with CD40 Deficiency. J Clin Immunol. 2023;44(1):17.
20. Meng X, Yang B, Suen WC. Prospects for modulating the CD40/CD40L pathway in the therapy of the hyper-IgM syndrome. Innate Immun. 2018;24(1):4-10.
21. Ambruso DR, Knall C, Abell AN, Panepinto J, Kurkchubasche A, Thurman G, et al. Human neutrophil immunodeficiency syndrome is associated with an inhibitory Rac2 mutation. Proc Natl Acad Sci U S A. 2000;97(9):4654-9.
22. Hsu AP, Donkó A, Arrington ME, Swamydas M, Fink D, Das A, et al. Dominant activating RAC2 mutation with lymphopenia, immunodeficiency, and cytoskeletal defects. Blood. 2019;133(18):1977-88.
23. Alkhairy OK, Rezaei N, Graham RR, Abolhassani H, Borte S, Hultenby K, et al. RAC2 loss-of-function mutation in 2 siblings with characteristics of common variable immunodeficiency. J Allergy Clin Immunol. 2015;135(5):1380-4.e1-5.
24. Donkó A, Sharapova SO, Kabat J, Ganesan S, Hauck F, Marois L, et al. Clinical and functional spectrum of RAC2-related immunodeficiency. Blood. 2024.
25. Lougaris V, Baronio M, Gazzurelli L, Benvenuto A, Plebani A. RAC2 and primary human immune deficiencies. Journal of Leukocyte Biology. 2020;108(2):687-96.
26. Stray-Pedersen A, Backe PH, Sorte HS, Mørkrid L, Chokshi NY, Erichsen HC, et al. PGM3 mutations cause a congenital disorder of glycosylation with severe immunodeficiency and skeletal dysplasia. Am J Hum Genet. 2014;95(1):96-107.
27. Sassi A, Lazaroski S, Wu G, Haslam SM, Fliegauf M, Mellouli F, et al. Hypomorphic homozygous mutations in phosphoglucomutase 3 (PGM3) impair immunity and increase serum IgE levels. J Allergy Clin Immunol. 2014;133(5):1410-9, 9.e1-13.
28. Zhang Y, Yu X, Ichikawa M, Lyons JJ, Datta S, Lamborn IT, et al. Autosomal recessive phosphoglucomutase 3 (PGM3) mutations link glycosylation defects to atopy, immune deficiency, autoimmunity, and neurocognitive impairment. J Allergy Clin Immunol. 2014;133(5):1400-9, 9.e1-5.
29. Engelhardt KR, McGhee S, Winkler S, Sassi A, Woellner C, Lopez-Herrera G, et al. Large deletions and point mutations involving the dedicator of cytokinesis 8 (DOCK8) in the autosomal-recessive form of hyper-IgE syndrome. J Allergy Clin Immunol. 2009;124(6):1289-302.e4.
30. Zhang Q, Davis JC, Lamborn IT, Freeman AF, Jing H, Favreau AJ, et al. Combined immunodeficiency associated with DOCK8 mutations. N Engl J Med. 2009;361(21):2046-55.

31. Wilkie H, Timilshina M, Rahmayanti S, Das M, Pelovitz T, Geha RS. DOCK8 is essential for neutrophil mediated clearance of cutaneous S. aureus infection. Clin Immunol. 2023;254:109681.
32. Keles S, Charbonnier LM, Kabaleeswaran V, Reisli I, Genel F, Gulez N, et al. Dedicator of cytokinesis 8 regulates signal transducer and activator of transcription 3 activation and promotes T(H)17 cell differentiation. J Allergy Clin Immunol. 2016;138(5):1384-94.e2.
33. Woellner C, Gertz EM, Schäffer AA, Lagos M, Perro M, Glocker EO, et al. Mutations in STAT3 and diagnostic guidelines for hyper-IgE syndrome. J Allergy Clin Immunol. 2010;125(2):424-32.e8.
34. Milner JD, Brenchley JM, Laurence A, Freeman AF, Hill BJ, Elias KM, et al. Impaired T(H)17 cell differentiation in subjects with autosomal dominant hyper-IgE syndrome. Nature. 2008;452(7188):773-6.
35. Holland SM, DeLeo FR, Elloumi HZ, Hsu AP, Uzel G, Brodsky N, et al. STAT3 mutations in the hyper-IgE syndrome. N Engl J Med. 2007;357(16):1608-19.
36. Minegishi Y, Saito M, Tsuchiya S, Tsuge I, Takada H, Hara T, et al. Dominant-negative mutations in the DNA-binding domain of STAT3 cause hyper-IgE syndrome. Nature. 2007;448(7157):1058-62.
37. Farmand S, Kremer B, Häffner M, Pütsep K, Bergman P, Sundin M, et al. Eosinophilia and reduced STAT3 signaling affect neutrophil cell death in autosomal-dominant Hyper-IgE syndrome. Eur J Immunol. 2018;48(12):1975-88.
38. Myles IA, Anderson ED, Earland NJ, Zarember KA, Sastalla I, Williams KW, et al. TNF overproduction impairs epithelial staphylococcal response in hyper IgE syndrome. J Clin Invest. 2018;128(8):3595-604.
39. Minegishi Y, Saito M, Nagasawa M, Takada H, Hara T, Tsuchiya S, et al. Molecular explanation for the contradiction between systemic Th17 defect and localized bacterial infection in hyper-IgE syndrome. J Exp Med. 2009;206(6):1291-301.
40. Frey-Jakobs S, Hartberger JM, Fliegauf M, Bossen C, Wehmeyer ML, Neubauer JC, et al. ZNF341 controls STAT3 expression and thereby immunocompetence. Sci Immunol. 2018;3(24).
41. Béziat V, Li J, Lin JX, Ma CS, Li P, Bousfiha A, et al. A recessive form of hyper-IgE syndrome by disruption of ZNF341-dependent STAT3 transcription and activity. Sci Immunol. 2018;3(24).
42. Béziat V, Tavernier SJ, Chen YH, Ma CS, Materna M, Laurence A, et al. Dominant-negative mutations in human IL6ST underlie hyper-IgE syndrome. J Exp Med. 2020;217(6).
43. Schwerd T, Twigg SRF, Aschenbrenner D, Manrique S, Miller KA, Taylor IB, et al. A biallelic mutation in IL6ST encoding the GP130 co-receptor causes immunodeficiency and craniosynostosis. J Exp Med. 2017;214(9):2547-62.
44. Shahin T, Aschenbrenner D, Cagdas D, Bal SK, Conde CD, Garncarz W, et al. Selective loss of function variants in IL6ST cause Hyper-IgE syndrome with distinct impairments of T-cell phenotype and function. Haematologica. 2019;104(3):609-21.
45. Chen YH, Grigelioniene G, Newton PT, Gullander J, Elfving M, Hammarsjö A, et al. Absence of GP130 cytokine receptor signaling causes extended Stüve-Wiedemann syndrome. J Exp Med. 2020;217(3).
46. Spencer S, Köstel Bal S, Egner W, Lango Allen H, Raza SI, Ma CA, et al. Loss of the interleukin-6 receptor causes immunodeficiency, atopy, and abnormal inflammatory responses. J Exp Med. 2019;216(9):1986-98.
47. Minegishi Y, Saito M, Morio T, Watanabe K, Agematsu K, Tsuchiya S, et al. Human tyrosine kinase 2 deficiency reveals its requisite roles in multiple cytokine signals involved in innate and acquired immunity. Immunity. 2006;25(5):745-55.
48. Kreins AY, Ciancanelli MJ, Okada S, Kong XF, Ramírez-Alejo N, Kilic SS, et al. Human TYK2 deficiency: Mycobacterial and viral infections without hyper-IgE syndrome. J Exp Med. 2015;212(10):1641-62.
49. Kilic SS, Hacimustafaoglu M, Boisson-Dupuis S, Kreins AY, Grant AV, Abel L, Casanova JL. A patient with tyrosine kinase 2 deficiency without hyper-IgE syndrome. J Pediatr. 2012;160(6):1055-7.
50. Ogishi M, Arias AA, Yang R, Han JE, Zhang P, Rinchai D, et al. Impaired IL-23-dependent induction of IFN-γ underlies mycobacterial disease in patients with inherited TYK2 deficiency. J Exp Med. 2022;219(10).
51. Lyons JJ, Liu Y, Ma CA, Yu X, O'Connell MP, Lawrence MG, et al. ERBIN deficiency links STAT3 and TGF-β pathway defects with atopy in humans. J Exp Med. 2017;214(3):669-80.
52. Toubiana J, Okada S, Hiller J, Oleastro M, Lagos Gomez M, Aldave Becerra JC, et al. Heterozygous STAT1 gain-of-function mutations underlie an unexpectedly broad clinical phenotype. Blood. 2016;127(25):3154-64.
53. Liu L, Okada S, Kong XF, Kreins AY, Cypowyj S, Abhyankar A, et al. Gain-of-function human STAT1 mutations impair IL-17 immunity and underlie chronic mucocutaneous candidiasis. J Exp Med. 2011;208(8):1635-48.
54. van Zelm MC, Bosco JJ, Aui PM, De Jong S, Hore-Lacy F, O'Hehir RE, et al. Impaired STAT3-Dependent Upregulation of IL2Rα in B Cells of a Patient With a STAT1 Gain-of-Function Mutation. Front Immunol. 2019;10:768.
55. Picard C, Casanova JL, Puel A. Infectious diseases in patients with IRAK-4, MyD88, NEMO, or IκBα deficiency. Clin Microbiol Rev. 2011;24(3):490-7.
56. Bucciol G, Moens L, Bosch B, Bossuyt X, Casanova JL, Puel A, Meyts I. Lessons learned from the study of human inborn errors of innate immunity. J Allergy Clin Immunol. 2019;143(2):507-27.
57. Israel L, Wang Y, Bulek K, Della Mina E, Zhang Z, Pedergnana V, et al. Human Adaptive Immunity Rescues an Inborn Error of Innate Immunity. Cell. 2017;168(5):789-800.e10.
58. Picard C, von Bernuth H, Ghandil P, Chrabieh M, Levy O, Arkwright PD, et al. Clinical features and outcome of patients with IRAK-4 and MyD88 deficiency. Medicine (Baltimore). 2010;89(6):403-25.
59. Abu-Humaidan AH, Elvén M, Sonesson A, Garred P, Sørensen OE. Persistent Intracellular Staphylococcus aureus in Keratinocytes Lead to Activation of the Complement System with Subsequent Reduction in the Intracellular Bacterial Load. Front Immunol. 2018;9:396.
60. Loh JM, Aghababa H, Proft T. Eluding the immune system's frontline defense: Secreted complement evasion factors of pathogenic Gram-positive cocci. Microbiol Res. 2023;277:127512.
61. Ram S, Lewis LA, Rice PA. Infections of people with complement deficiencies and patients who have undergone splenectomy. Clin Microbiol Rev. 2010;23(4):740-80.
62. Brodszki N, Frazer-Abel A, Grumach AS, Kirschfink M, Litzman J, Perez E, et al. European Society for Immunodeficiencies (ESID) and European Reference Network on Rare Primary Immunodeficiency, Autoinflammatory and Autoimmune Diseases (ERN RITA) Complement Guideline: Deficiencies, Diagnosis, and Management. J Clin Immunol. 2020;40(4):576-91.
63. Spaan AN, Neehus AL, Laplantine E, Staels F, Ogishi M, Seeleuthner Y, et al. Human OTULIN haploinsufficiency impairs cell-intrinsic immunity to staphylococcal α-toxin. Science. 2022;376(6599):eabm6380.
64. Nguyen MH, Kauffman CA, Goodman RP, Squier C, Arbeit RD, Singh N, et al. Nasal carriage of and infection with Staphylococcus aureus in HIV-infected patients. Ann Intern Med. 1999;130(3):221-5.
65. Cole J, Popovich K. Impact of community-associated methicillin resistant Staphylococcus aureus on HIV-infected patients. Curr HIV/AIDS Rep. 2013;10(3):244-53.

66. Moylett EH, Shearer WT. HIV: clinical manifestations. J Allergy Clin Immunol. 2002;110(1):3-16.
67. Reed C, Kallen AJ, Patton M, Arnold KE, Farley MM, Hageman J, Finelli L. Infection with community-onset Staphylococcus aureus and influenza virus in hospitalized children. Pediatr Infect Dis J. 2009;28(7):572-6.
68. Rynda-Apple A, Robinson KM, Alcorn JF. Influenza and Bacterial Superinfection: Illuminating the Immunologic Mechanisms of Disease. Infect Immun. 2015;83(10):3764-70.
69. Widmer AF, Kern WV, Roth JA, Dettenkofer M, Goetting T, Bertz H, Theilacker C. Early versus late onset bloodstream infection during neutropenia after high-dose chemotherapy for hematologic malignancy. Infection. 2019;47(5):837-45.
70. Spinardi JR, Berea R, Orioli PA, Gabriele MM, Navarini A, Marques MT, et al. Enterococcus spp. and S. aureus colonization in neutropenic febrile children with cancer. Germs. 2017;7(2):61-72.
71. McNeil JC, Hulten KG, Kaplan SL, Mahoney DH, Mason EO. Staphylococcus aureus infections in pediatric oncology patients: high rates of antimicrobial resistance, antiseptic tolerance and complications. Pediatr Infect Dis J. 2013;32(2):124-8.
72. Puel A, Picard C, Lorrot M, Pons C, Chrabieh M, Lorenzo L, et al. Recurrent staphylococcal cellulitis and subcutaneous abscesses in a child with autoantibodies against IL-6. J Immunol. 2008;180(1):647-54.
73. Nanki T, Onoue I, Nagasaka K, Takayasu A, Ebisawa M, Hosoya T, et al. Suppression of elevations in serum C reactive protein levels by anti-IL-6 autoantibodies in two patients with severe bacterial infections. Ann Rheum Dis. 2013;72(6):1100-2.
74. Bloomfield M, Parackova Z, Cabelova T, Pospisilova I, Kabicek P, Houstkova H, Sediva A. Anti-IL6 Autoantibodies in an Infant With CRP-Less Septic Shock. Front Immunol. 2019;10:2629.
75. Nguyen MT, Pødenphant J, Ravn P. Three cases of severely disseminated Staphylococcus aureus infection in patients treated with tocilizumab. BMJ Case Rep. 2013;2013.
76. Bari SF, Khan A, Lawson T. C reactive protein may not be reliable as a marker of severe bacterial infection in patients receiving tocilizumab. BMJ Case Rep. 2013;2013.
77. Aeschlimann FA, Dumaine C, Wörner A, Mouy R, Wouters C, Melki I, et al. Serious adverse events in children with juvenile idiopathic arthritis and other rheumatic diseases on tocilizumab - a real-world experience. Semin Arthritis Rheum. 2020;50(4):744-8.
78. Blanchard AC, Waters VJ. Microbiology of Cystic Fibrosis Airway Disease. Semin Respir Crit Care Med. 2019;40(6):727-36.
79. Souche A, Vandenesch F, Doléans-Jordheim A, Moreau K. How Staphylococcus aureus and Pseudomonas aeruginosa Hijack the Host Immune Response in the Context of Cystic Fibrosis. Int J Mol Sci. 2023;24(7).
80. Chen Q, Shen Y, Zheng J. A review of cystic fibrosis: Basic and clinical aspects. Animal Model Exp Med. 2021;4(3):220-32.
81. Shahrokh S, Tabatabaee A, Yazdi M, Siavash M. Proportion of toxin and non-toxin virulence factors of Staphylococcus aureus isolates from diabetic foot infection: a systematic review and meta-analysis. BMC Microbiol. 2024;24(1):1.
82. Genito CJ, Darwitz BP, Greenwald MA, Wolfgang MC, Thurlow LR. Hyperglycemia potentiates increased Staphylococcus aureus virulence and resistance to growth inhibition by Pseudomonas aeruginosa. Microbiol Spectr. 2023;11(6):e0229923.
83. Fajans SS, Bell GI, Polonsky KS. Molecular mechanisms and clinical pathophysiology of maturity-onset diabetes of the young. N Engl J Med. 2001;345(13):971-80.
84. Quinn M, Fleischman A, Rosner B, Nigrin DJ, Wolfsdorf JI. Characteristics at diagnosis of type 1 diabetes in children younger than 6 years. J Pediatr. 2006;148(3):366-71.
85. Menne EN, Sonabend RY, Mason EO, Lamberth LB, Hammerman WA, Minard CG, et al. Staphylococcus aureus infections in pediatric patients with diabetes mellitus. J Infect. 2012;65(2):135-41.
86. Leistner R, Hanitsch LG, Krüger R, Lindner AK, Stegemann MS, Nurjadi D. Skin Infections Due to Panton-Valentine Leukocidin-Producing S. Aureus. Dtsch Arztebl Int. 2022;119(45):775-84.
87. Shilo N, Quach C. Pulmonary infections and community associated methicillin resistant Staphylococcus aureus: a dangerous mix? Paediatr Respir Rev. 2011;12(3):182-9.
